# The Interplay of Synaptic Plasticity and Scaling Enables Self-Organized Formation and Allocation of Multiple Memory Representations

**DOI:** 10.3389/fncir.2020.541728

**Published:** 2020-10-07

**Authors:** Johannes Maria Auth, Timo Nachstedt, Christian Tetzlaff

**Affiliations:** ^1^Department of Computational Neuroscience, Third Institute of Physics, Georg-August-Universität, Göttingen, Germany; ^2^Bernstein Center for Computational Neuroscience, Göttingen, Germany

**Keywords:** memory allocation, memory formation, synaptic plasiticity, synaptic scaling, network dynamic

## Abstract

It is commonly assumed that memories about experienced stimuli are represented by groups of highly interconnected neurons called cell assemblies. This requires allocating and storing information in the neural circuitry, which happens through synaptic weight adaptations at different types of synapses. In general, memory allocation is associated with synaptic changes at feed-forward synapses while memory storage is linked with adaptation of recurrent connections. It remains, however, largely unknown how memory allocation and storage can be achieved and the adaption of the different synapses involved be coordinated to allow for a faithful representation of *multiple* memories without disruptive interference between them. In this theoretical study, by using network simulations and phase space analyses, we show that the interplay between long-term synaptic plasticity and homeostatic synaptic scaling organizes simultaneously the adaptations of feed-forward and recurrent synapses such that a new stimulus forms a new memory and where different stimuli are assigned to distinct cell assemblies. The resulting dynamics can reproduce experimental *in-vivo* data, focusing on how diverse factors, such as neuronal excitability and network connectivity, influence memory formation. Thus, the here presented model suggests that a few fundamental synaptic mechanisms may suffice to implement memory allocation and storage in neural circuitry.

## 1. Introduction

Learning and memorizing information about the environment over long time scales is a vital function of neural circuits of living beings. For this, different elements of a neural circuit—the neurons and synapses—have to coordinate themselves to accurately form, organize, and allocate internal long-term representations of the different pieces of information received. While many processes and dynamics of the single elements are well-documented, their coordination remains obscure. How do neurons and synapses self-organize to form functional, stable, and distinguishable memory representations? Moreover, what mechanisms underlie the self-organized coordination yielding such representations?

This theoretical study identifies three distinct properties of long-term synaptic dynamics to allow for a robust coordination of neurons and synapses during the self-organized formation of internal representations. In contrast to the current hypothesis, which assumes that solely long-term synaptic plasticity serves as sufficient mechanism (Martin et al., 2000; Barbieri and Brunel, 2008; Palm et al., 2014; Takeuchi et al., 2014), our analysis indicates that synaptic plasticity has to interact with the mechanism so-called synaptic scaling, which adapts synaptic dynamics in a homeostatic manner (Turrigiano et al., 1998; Tetzlaff et al., 2011; Hengen et al., 2013). This interaction of plasticity mechanisms, determining the dynamics of individual synapses, comprises all three required properties and results in an input-dependent circuit dynamic, which yields the reliable organization of internal representations of environmental information.

As discussed in the following, to understand the coordination of neurons and synapses underlying the organization of internal representations, we have to investigate two memory processes acting together: memory formation and allocation.

The reliable *formation* of memory, hence of internal stimulus representations in neural circuits, is explained by the Hebbian hypothesis. In brief, the Hebbian hypothesis (James, 1890; Konorski, 1948; Hebb, 1949; Palm et al., 2014; Holtmaat and Caroni, 2016) states that, when a neural circuit receives a new piece of information, the corresponding stimulus activates a group of neurons and, via activity-dependent long-term synaptic plasticity (Bliss and Lomo, 1973; Levy and Steward, 1983; Martin et al., 2000; Malenka and Bear, 2004), adapts the weights or efficacies of synapses between these neurons. This adaptation remodels the activated group of neurons to a strongly interconnected group of neurons called Hebbian cell assembly (HA). This newly formed HA serves as an internal long-term representation (long-term memory) of the corresponding stimulus (Hebb, 1949; Palm et al., 2014; Holtmaat and Caroni, 2016). Recall of this memory translates into the activation of the respective HA. In order to recognize similar pieces of information, similar stimuli also have to be able to activate the corresponding HA. This is enabled by the strong recurrent interconnections between HA-neurons resulting in pattern completion (Hunsaker and Kesner, 2013; Palm et al., 2014). Several experimental and theoretical studies have investigated the formation and recall of HAs given synaptic plasticity (Hopfield, 1982, 1984; Amit et al., 1985, 1994; Tsodyks and Feigelman, 1988; Buzsaki, 2010; Brunel, 2016; Holtmaat and Caroni, 2016). Recent theoretical studies already indicate that, in addition to synaptic plasticity, homeostatic mechanisms such as synaptic scaling are required to keep the neural circuit in a functional regime (Tetzlaff et al., 2013, 2015; Litwin-Kumar and Doiron, 2014; Zenke et al., 2015). However, all the above-mentioned studies investigate the coordination of synaptic and neuronal dynamics within the group of neurons (memory formation), but they do not consider the dynamics determining which neurons are recruited to form this group, or rather why is the stimulus allocated to this specific group of neurons and not to others (memory allocation; Rogerson et al., 2014).

The *allocation* of stimuli to their internal representations requires that the synapses from the neurons encoding the stimulus to the neurons encoding the internal representation have to be adjusted accordingly. Considering only these types of synapses (“feed-forward synapses”), several studies show that long-term synaptic plasticity yields the required adjustments of synaptic weights (Willshaw et al., 1969; Adelsberger-Mangan and Levy, 1992; Knoblauch et al., 2010; Babadi and Sompolinsky, 2014; Kastellakis et al., 2016; Choi et al., 2018) such that each stimulus is mapped to its corresponding group of neurons. However, theoretical studies (Sullivan and de Sa, 2006; Stevens et al., 2013), which investigate the formation of input-maps in the visual cortex (Kohonen, 1982; Obermayer et al., 1990), show that the stable mapping or allocation of stimuli onto a neural circuit requires, in addition to synaptic plasticity, homeostatic mechanisms such as synaptic scaling. Note that all these studies focus on the allocation of stimuli to certain groups of neurons; however, they do not consider the dynamics of the synapses within these groups (“recurrent synapses”).

Thus, up to now, it remains unclear how a neural circuit coordinates in a self-organized manner the synaptic and neuronal dynamics underlying the reliable allocation of stimuli to neurons with the simultaneous dynamics underlying the proper formation of memory representations. If these two memory processes are not tightly coordinated, the neural system could show awkward, undesired dynamics: On the one hand, memory allocation could map a stimulus to a group of unconnected neurons, which impedes the formation of a proper HA. On the other hand, the formation of a HA could bias the dynamics of allocation such that multiple stimuli are mapped onto the same HA disrupting the ability to discriminate between these stimuli.

In this theoretical study, we show in a network model that long-term synaptic plasticity (Hebb, 1949; Abbott and Nelson, 2000; Gerstner and Kistler, 2002) together with the slower, homeostatic processes of synaptic scaling (Turrigiano et al., 1998; Turrigiano and Nelson, 2004; Tetzlaff et al., 2011; Hengen et al., 2013) leads to the self-organized coordination of synaptic weight changes at feed-forward and recurrent synapses. Throughout this study, the system should memorize two independent stimuli of arbitrary modality illustrating a general aspect of explicit memory. The synaptic changes of the recurrent synapses yields the reliable formation of HAs. In parallel, the synaptic changes of the feed-forward synapses links the newly formed HA with the corresponding stimulus-transmitting neurons without interrupting already learned ones assuring the allocation of HAs. The model reproduces *in-vivo* experimental data and provides testable predictions. Furthermore, the analysis of a population model, capturing the main features of the network dynamics, allows us to determine three generic properties of the interaction between synaptic plasticity and scaling, which enable the formation and allocation of memory representations in a reliable manner. These properties of synaptic adaptation are that (i) synaptic weights between two neurons with highly-correlated activities are strengthened (homosynaptic potentiation), (ii) synaptic weights between two neurons with weakly-correlated activities are lowered (heterosynaptic depression), and (iii) the time scale of synaptic weight changes are regulated by the post-synaptic activity level.

## 2. Materials and Methods

### 2.1. Numerical Simulations

**Figure 1:**
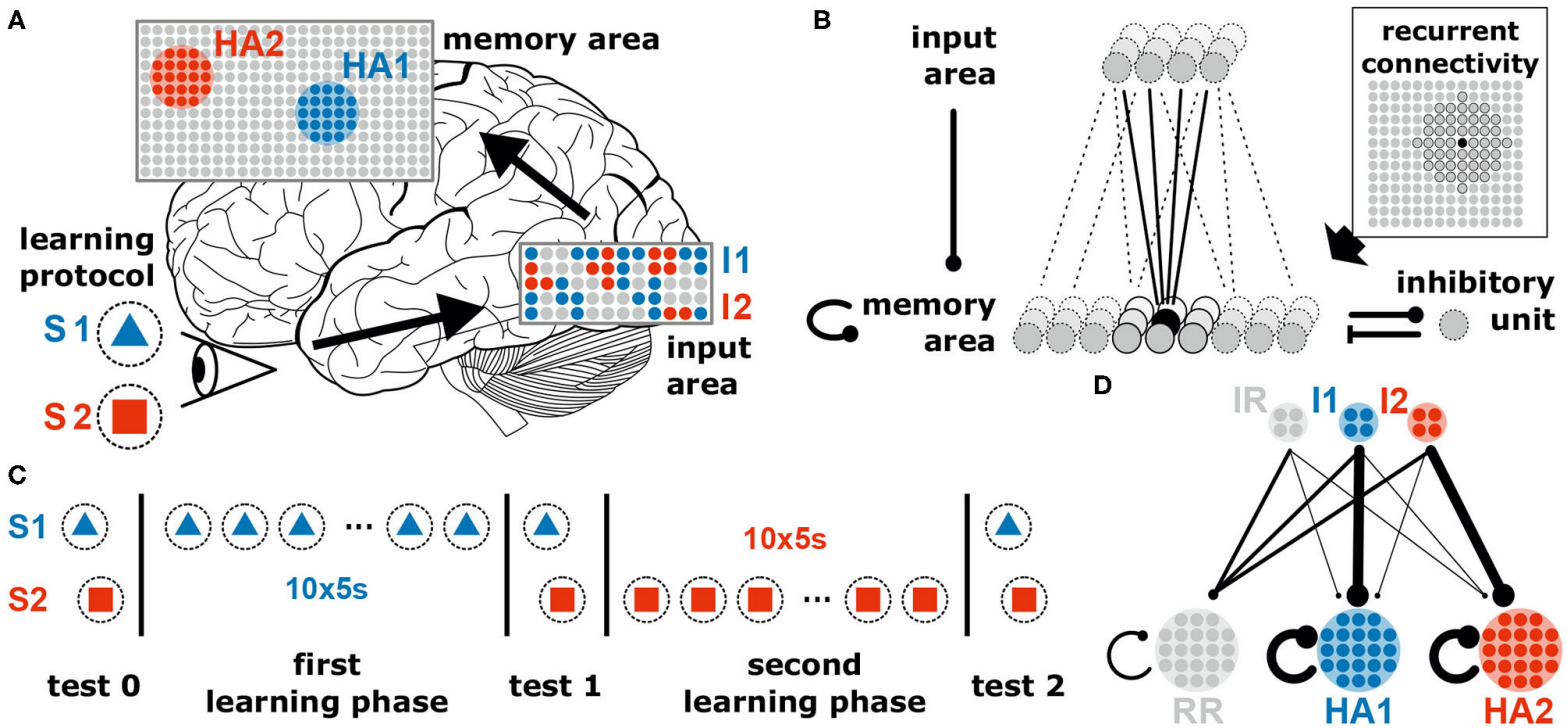
The neural system to investigate the coordination of synaptic and neuronal dynamics underlying the allocation and formation of memories consists of two areas receiving external stimuli. **(A)** The neural system receives different external stimuli (e.g., a blue triangle, S1, or a red square, S2) yielding the activation of subsets of neurons (colored dots) in the input (I1 and I2) and memory area (HA1 and HA2; brain image by James.mcd.nz under license CC BY-SA 4.0). **(B)** Each excitatory neuron in the memory area receives inputs from a random subset of excitatory neurons being in the input area, from its neighboring neurons of the memory area (indicated by the dark gray units in the inset) and from a global inhibitory unit. All synapses between excitatory neurons are plastic regarding the interplay of long-term synaptic plasticity and synaptic scaling. **(C)** Throughout this study, we consider two learning phases during each a specific stimulus is repetitively presented. In addition, test phases are considered with stopped synaptic dynamics for analyses. **(D)** Schematic illustration of the average synaptic structure ensuring a proper function of the neural system. This structure should result from the neuronal and synaptic dynamics in conjunction with the stimulation protocol. IR and RR represent populations of remaining neurons being not directly related to the dynamics of memory formation and allocation. Details see main text.

The neuronal system considered in this study consists of a recurrently connected neuronal network (“memory area”) receiving (environmental) inputs from an “input area” via plastic feed-forward synapses (Figures 1A,B). The system consists of 936 excitatory neurons (36 in input area, 900 in memory area) and a single inhibitory unit. The inhibitory unit describes a population of inhibitory neurons which are connected to the excitatory neurons in an all-to-all manner. Given the long time scales considered in this study, all neurons are described by a rate-coded leaky integrator model. The memory area is arranged as a quadratic neural grid of 30 × 30 units. Each neuron within the grid receives excitatory connections from four randomly chosen input neurons. In addition, it is recurrently connected to its circular neighborhood of radius four (measured in neuronal units; for visualization see Figure 1B and Supplementary Figure S1) and to the global inhibitory unit. Initially, recurrent synaptic weights of existing connections equal to 0.25 · ŵ^rec^ and feed-forward synaptic weights are drawn from a uniform distribution {0, 0.7 · ŵ^ff^} (${ŵ}^{\text{rec}}=\sqrt{\left(\kappa^{\text{rec}}\alpha^{2}\right)/\left(\alpha -F^{\text{T}}\right)}\approx 77.5$ and ${ŵ}^{\text{ff}}=\sqrt{\left(\kappa^{\text{ff}}\alpha \cdot 130\right)/\left(\alpha -F^{\text{T}}\right)}\approx 306.1$). Connections to and from the inhibitory neuron are at fixed and homogeneous weight (for detailed values see Table 1).

**Table 1:** Model parameters: variable, descriptions, and used values.

**Variable**	**Description**	**Value**
τ	Membrane time constant (memory area)	0.01
*R*	Membrane resistance (memory area)	1/11
*N*^M^	Number of neurons in memory area	900
*N*^I^	Number of neurons in input area	36
*I*_k_	Input rate	{0,130}
α	Maximum firing rate	100
β	Sigmoid steepness	0.05
ϵ	Sigmoid inflection point	130
μ	Plasticity time constant	1/15
*F*^T^	Target firing rate	0.1
κ^rec^	Scaling time constant (recurrent)	60
κ^ff^	Scaling time constant (feed-forward)	720
τ_inh_	Membrane time constant (inhibitory unit)	0.02
*R*_inh_	Membrane resistance (inhibitory unit)	1
*w*_inh,i_	Synaptic weight to inhibitory unit	0.6
*w*_i,inh_	Synaptic weight from inhibitory unit	1,200

#### 2.1.1. Neuron Model

The membrane potential *u*_i_ of each excitatory neuron *i* in the memory area is described as follows:

(1) $$\begin{array}{r} \frac{\text{d}u_{\text{i}}}{\text{d}t}=-\frac{u_{\text{i}}}{\tau }+R\left(\overset{N^{\text{M}}}{\underset{\text{j}}{\sum }}w_{\text{i,j}}^{\text{rec}}F_{\text{j}}+w_{\text{i,inh}}F_{\text{inh}}+\overset{N^{\text{I}}}{\underset{\text{k}}{\sum }}w_{\text{i,k}}^{\text{ff}}I_{\text{k}}\right), \end{array}$$

with the membrane time constant τ, membrane resistance *R*, number of neurons in the memory area *N*^M^, number of neurons in the input area *N*^I^, and firing rate *I*_k_ of input neuron *k*. The membrane potential is converted into a firing rate *F*_i_ by a sigmoidal transfer function with maximal firing rate α, steepness β and inflection point ϵ:

(2) $$\begin{array}{r} F_{\text{i}}\left(u_{\text{i}}\right)=\frac{\alpha }{1+\exp\left(\beta \left(\epsilon -u_{\text{i}}\right)\right)}. \end{array}$$

The global inhibitory unit is also modeled as a rate-coded leaky integrator receiving inputs from all neurons of the memory area. Its membrane potential *u*_inh_ follows the differential equation 3 with inhibitory membrane time scale τ_inh_ and resistance *R*_inh_. The potential is converted into a firing rate *F*_inh_ by a sigmoidal transfer function (Equation 4):

(3) $$\begin{array}{r} \frac{\text{d}u_{\text{inh}}}{\text{d}t}=-\frac{u_{\text{inh}}}{\tau_{\text{inh}}}+R_{\text{inh}}\cdot \overset{N^{\text{M}}}{\underset{\text{i}}{\sum }}w_{\text{inh,i}}F_{\text{i}}, \end{array}$$

(4) $$\begin{array}{r} F_{\text{inh}}\left(u_{\text{inh}}\right)=\frac{\alpha }{1+\exp\left(\beta \left(\epsilon -u_{\text{inh}}\right)\right)}. \end{array}$$

As the neurons in the input area form the stimuli, their output activation is set manually. Thus no further description is needed.

#### 2.1.2. Synaptic Dynamics

The weight changes of the excitatory feed-forward (Equation 5) and recurrent synapses (Equation 6) are determined by the combined learning rule of conventional Hebbian synaptic plasticity and synaptic scaling with time constants μ^ff^, μ^rec^, κ^ff^, κ^rec^, and target firing rate *F*^T^. The differential equation for the synaptic weight of a feed-forward connection $w_{\text{i,k}}^{\text{ff}}$ from input neuron *k* ∈ {0, ⋯ , *N*^I^} to memory neuron *i* ∈ {0, ⋯ , *N*^M^} is:

(5) $$\begin{array}{r} \frac{\text{d}w_{\text{i,k}}^{\text{ff}}}{\text{d}t}=\mu^{\text{ff}}\left(F_{\text{i}}I_{\text{k}}+{\left(\kappa^{\text{ff}}\right)}^{-1}\cdot \left(F^{\text{T}}-F_{\text{i}}\right)\cdot {\left(w_{\text{i,k}}^{\text{ff}}\right)}^{2}\right)\cdot c_{\text{i,k}}^{\text{ff}}. \end{array}$$

The dynamics of the synaptic weight of a recurrent connection $w_{\text{i,j}}^{\text{rec}}$ from memory neuron *j* ∈ {0, ⋯ , *N*^M^} to memory neuron *i* ∈ {0, ⋯ , *N*^M^} is determined by:

(6) $$\begin{array}{r} \frac{\text{d}w_{\text{i,j}}^{\text{rec}}}{\text{d}t}=\mu^{\text{rec}}\left(F_{\text{i}}F_{\text{j}}+{\left(\kappa^{\text{rec}}\right)}^{-1}\cdot \left(F^{\text{T}}-F_{\text{i}}\right)\cdot {\left(w_{\text{i,j}}^{\text{rec}}\right)}^{2}\right)\cdot c_{\text{i,j}}^{\text{rec}}. \end{array}$$

$c_{\text{i,k}}^{\text{ff}}$ and $c_{\text{i,k}}^{\text{ff}}$ are the entries in the feed-forward and recurrent connectivity matrices of value 1, if the connection exists, or otherwise of value 0.

In both equations the first summand on the right hand side describes correlation-based Hebbian synaptic plasticity while the second summand formalizes the dynamics of synaptic scaling. Synaptic scaling alone drives the synaptic weights in a homeostatic manner such that the neuronal firing rate *F*_i_ reaches its target rate *F*^T^. As shown in previous studies (Tetzlaff et al., 2011, 2013, 2015; Yger and Gilson, 2015), although the homeostatic dynamics of scaling are significantly slower than the fast, divergent dynamics of Hebbian plasticity (κ^ff^, κ^rec^ >> 1), the interplay between both processes yields synaptic dynamics that remain in a reasonable regime. W.l.o.g., we consider that the overall time scale of the synaptic dynamics of feed-forward and recurrent connections is the same (μ^rec^ = μ^ff^ = μ; see Supplementary Figure S3). All other connections are considered to be non-plastic.

#### 2.1.3. Coding Framework

The differential equations have been solved numerically with the Euler method with a time step of 5 ms using Python 3.5.

#### 2.1.4. Simulation of Self-Organized Formation of Two HAs

The system undergoes two learning phases presenting two completely dissimilar input patterns I1 (first phase) and I2 (second phase). For input pattern I1 half of the input neurons are set to be active at 130 Hz whereas the other half remains inactive at 0 Hz and vice versa for input pattern I2. During a learning phase the respective pattern is presented 10 times for 5 s with a 1 s pause in between. Both learning phases are embraced by test phases in which plasticity is shut off and both patterns are presented for 0.5 s each to apply measures on the existing memory structures.

#### 2.1.5. Comparison to Experimental Data

The manipulation of the neuronal excitability has been done by adapting the value for ϵ in the transfer function of the neuron model, i.e., shifting its inflection point to lower (increased excitability) or higher (decreased excitability) values. Similar to the methods used in experiments (Yiu et al., 2014), we manipulated a sub-population of neurons within a randomly chosen circular area in the memory area (about 10% of the network). The relative recruitment factor is the relation of recruitment probabilities for manipulated and control neurons averaged over 100 repetitions.

### 2.2. Population Model

We consider two non-overlapping populations of *N* excitatory neurons and one inhibitory unit. The state of every population *i* ∈ {1, 2} is determined by its mean membrane potential ū_*i*_, its mean recurrent synaptic weight ${\bar{w}}_{i}^{\text{rec}}$ between neurons of the population, and the mean weight ${\bar{w}}_{i}^{\text{ff}}$ of feed-forward synapses projecting signals from the currently active input onto the population. We assume that the two populations interact solely through the inhibitory unit whose state is given by its membrane potential *u*_inh_. Thus, the dynamics of the model is described by a set of nine differential equations (see following section). To obtain its equilibria, we analytically derive the nullclines ${ū}_{1}^{*}\left({ū}_{2}^{*}\right)$ and ${ū}_{2}^{*}\left({ū}_{1}^{*}\right)$ and numerically determine their intersections. The stability of an equilibrium is obtained from the sign of the eigenvalues of the system's Jacobi Matrix. For analyzing in which regimes population 1 and population 2 are assigned to an input stimulus as a function of the initial synaptic weights (**Figures 5E**, **6**), we initialize the system with the given combination of feed-forward and recurrent average synaptic weights and ū_1_ = ū_2_ = ū_inh_ = 0, simulate it for 100 s, and assess which of the two populations is active. For further details and parameter values see Supplementary Material.

#### 2.2.1. Derivation of Population Model

The two excitatory populations in the population model are described by their mean membrane potentials ū_*i*_, *i* ∈ {1, 2}, *k* ∈ {A, B}:

(7) $$\begin{array}{r} \frac{\text{d}{ū}_{i}}{\text{d}t}=-\frac{{ū}_{i}}{\tau }+R\left({\bar{n}}^{\text{rec}}{\bar{w}}_{i}^{\text{rec}}{\bar{F}}_{i}+w_{i,\text{inh}}F_{\text{inh}}+\underset{k}{\sum }{\bar{n}}^{\text{ff}}{\bar{w}}_{ik}^{\text{ff}}{Ī}_{k}\right). \end{array}$$

Here, the time scale τ, the resistance *R* and the synaptic weight *w*_*i*, inh_ have the same value as in the network simulations. The average number ${\bar{n}}_{i}^{\text{rec}}$ of incoming recurrent connections from neurons within the population as well as the number of feed-forward synapses transmitting signals from active inputs to every neuron (${\bar{n}}^{\text{ff}}$) are taken from simulations (${\bar{n}}^{\text{rec}}=35$, Supplementary Figure S4C; *n*^ff^ = 2.3, Supplementary Figure S4B).

The membrane potential of the inhibitory population is given by

(8) $$\begin{array}{r} \frac{\text{d}u_{\text{inh}}}{\text{d}t}=-\frac{u_{\text{inh}}}{\tau_{\text{inh}}}+R_{\text{inh}}\left(w_{\text{inh},1}N{\bar{F}}_{1}+w_{\text{inh},2}N{\bar{F}}_{2}\right) \end{array}$$

with τ_inh_, *R*_inh_ and *w*_inh,1_ = *w*_inh,2_ corresponding to the respective values in the network simulations. The number *N* of neurons per population is adjusted to the HAs in the network simulation and chosen as *N* = 120 (Supplementary Figure S4A). The transfer function of the neurons within the population is the same as for individual neurons:

(9) $$\begin{array}{r} {\bar{F}}_{i}\left({ū}_{i}\right)=\frac{\alpha }{1+\exp\left(\beta \left(\epsilon -{ū}_{i}\right)\right)},i\in \left\{1,2,\text{inh}\right\}. \end{array}$$

The synaptic weight changes of recurrent and feed-forward synapses follow the interplay of conventional Hebbian synaptic plasticity and synaptic scaling (*i* ∈ {1, 2}):

(10) $$\begin{array}{r} \frac{\text{d}{\bar{w}}_{ik}^{\text{ff}}}{dt}=\mu \left({\bar{F}}_{i}{Ī}_{k}+{\left(\kappa^{\text{ff}}\right)}^{-1}\left(F^{\text{T}}-{\bar{F}}_{i}\right){\left({\bar{w}}_{ik}^{\text{ff}}\right)}^{2}\right), \end{array}$$

(11) $$\begin{array}{r} \frac{\text{d}{\bar{w}}_{i}^{\text{rec}}}{\text{d}t}=\mu \left({\bar{F}}_{i}^{2}+{\left(\kappa^{\text{rec}}\right)}^{-1}\left(F^{\text{T}}-{\bar{F}}_{i}\right){\left({\bar{w}}_{i}^{\text{rec}}\right)}^{2}\right). \end{array}$$

#### 2.2.2. Data Display

The population model applies the same combined learning rule as the numerical simulation. We thus consider the memorization process completed when the dynamic fixed point of synaptic plasticity is reached, i.e., synaptic plasticity and scaling compensate each other. In order to focus the reader's attention onto the populations dynamics as well as for illustrative reasons, we scale the explicit values of synaptic weights and activation in their depiction (**Figures 5B–E**, **6B,D** and Supplementary Figure S5) with the respective values of their dynamic fixed points (weights; ŵ^ff^, ŵ^rec^) or the maximum value (activation; α). Hence, their values simply range from 0 to 1.

#### 2.2.3. Recruitment Basins

For determining the recruitment basins (**Figures 5E**, **6**), we exploit the symmetry of the system and that, in general, only one of the two stimuli (S1 or S2) is active. Accordingly, we approximate the second, inactive input to zero and neglect the respective feed-forward synapses. The population model is integrated with the given initial values of the feed-forward and recurrent weights and ū_1_ = ū_2_ = ū_inh_ = 0 for 100s. At *t* = 100s, we evaluate which of the two populations is active. Table 2 provides the exact used initial values.

**Table 2:** Initial values for recruitment basin plots.

**Figure**	**ū_1_**	**ū_2_**	**ū_inh_**	${\bar{w}}_{1\text{A}}^{\text{ff}}$	${\bar{w}}_{2\text{A}}^{\text{ff}}$	${\bar{w}}_{1}^{\text{rec}}$	${\bar{w}}_{2}^{\text{rec}}$
2E left; 2E right^†^;							
3B top left, 3B top right^†^,	0	0	0	$0,\Delta w_{1\text{A}}^{\text{ff}},\ldots ,{ŵ}_{1\text{A}}^{\text{ff}}$	$0.35{ŵ}_{2\text{A}}^{\text{ff}}$	$0,\Delta w_{1}^{\text{rec}},\ldots ,{ŵ}_{1}^{\text{rec}}$	$0.25{ŵ}_{2}^{\text{rec}}$
3B bottom right^*^^†^,							
3D top left, 3D top right^†^							
3B bottom left^*^	0	0	0	$0,\Delta w_{1\text{A}}^{\text{ff}},\ldots ,{ŵ}_{1\text{A}}^{\text{ff}}$	$0.40{ŵ}_{2\text{A}}^{\text{ff}}$	$0,\Delta w_{1}^{\text{rec}},\ldots ,{ŵ}_{1}^{\text{rec}}$	$0.25{ŵ}_{2}^{\text{rec}}$
3D bottom left^*^	0	0	0	$0,\Delta w_{1\text{A}}^{\text{ff}},\ldots ,{ŵ}_{1\text{A}}^{\text{ff}}$	$1.00{ŵ}_{2\text{A}}^{\text{ff}}$	$0,\Delta w_{1}^{\text{rec}},\ldots ,{ŵ}_{1}^{\text{rec}}$	$1.00{ŵ}_{2}^{\text{rec}}$
3D bottom right^*^^†^	0	0	0	$0,\Delta w_{1\text{A}}^{\text{ff}},\ldots ,{ŵ}_{1\text{A}}^{\text{ff}}$	$0.00{ŵ}_{2\text{A}}^{\text{ff}}$	$0,\Delta w_{1}^{\text{rec}},\ldots ,{ŵ}_{1}^{\text{rec}}$	$1.00{ŵ}_{2}^{\text{rec}}$

$\Delta w_{1\text{A}}^{\text{ff}}=0.001{ŵ}_{1\text{A}}^{\text{ff}}$, $\Delta w_{1}^{\text{rec}}=0.001{ŵ}_{1}^{\text{rec}}$.

* *Using symmetry by commutating populations*.

† *Using symmetry by commutating inputs*.

## 3. Results

Throughout this study, our goal is to present a general model and its key mechanism that may underlie self-organized memory allocation and formation. We consider a neural system receiving environmental stimuli (sensory, pain, fear, etc.) that are processed and eventually memorized or encoded. In order to illustrate our experimental procedure, we choose an exemplary learning protocol for visual memory (Figure 1). Here, different stimuli such as geometrical shapes (e.g., a blue triangle S1 and a red square S2) are presented to the system, which then evoke certain activity patterns within an “input area” (e.g., blue pattern I1 evoked by S1). Each activity pattern, in turn, triggers via several random feed-forward synapses the activation of a subset of neurons in a recurrently connected “memory area.” For simplicity, all neurons of the system are considered to be excitatory; only a single all-to-all connected inhibitory unit (resembling an inhibitory population) in the memory area regulates its global activity level (Figure 1B). Furthermore, as we investigate here neuronal and synaptic dynamics happening on long time scales, we neglect the influence of single spikes and, thus, directly consider the dynamics of the neuronal firing rates (see section 2). All synapses between the excitatory neurons (feed-forward and recurrent) are adapted by activity-dependent long-term plasticity. Due to the recurrent connections, the memory area should robustly form internal representations of the environmental stimuli, while simultaneously the feed-forward synapses should provide an allocation of the stimuli (activity patterns in input area) onto the corresponding representations. In the following, we will show in a step-by-step approach that the interplay of long-term synaptic plasticity (here conventional Hebbian synaptic plasticity) with homeostatic synaptic scaling (see section 2; Tetzlaff et al., 2011, 2013; Nachstedt and Tetzlaff, 2017) coordinates synaptic changes such that proper formation and allocation of memory is ensured.

First, stimulus S1 is repetitively presented ten times (first learning phase; Figure 1C). Given this stimulation, the resulting synaptic adaptations of feed-forward and recurrent synapses should yield the proper formation of an internal representation indicating that the dynamics underlying memory allocation (changes of feed-forward synapses) does not impede the recurrent dynamics of HA-formation. After the formation of this representation, next, we repetitively present a different stimulus S2 (second learning phase). Due to this stimulation, the neural system should form another HA representing S2, which is independent of the first one. The proper formation of a second HA indicates that memory allocation is not biased by recurrent dynamics enabling a reliable discrimination between stimuli. Please note that we consider three test phases (Figure 1C), during which synaptic dynamics are fixed (separation of time scales), to enable the investigation of the resulting response dynamics of the circuit according to the different stimuli. Otherwise, the system is always plastic. In general, we expect that the neural system should form strongly interconnected groups of neurons according to each learning stimulus (memory formation; HA1 and HA2 in Figure 1D indicated by thicker lines) while remaining neurons in the memory area (RR) last weakly interconnected. In addition, the synapses from the neurons in the input area, which are activated by a specific stimulus (I1 and I2), to the corresponding HAs should have larger weights while all other feed-forward connections remain rather weak (memory allocation; I1 to HA1 and I2 to HA2).

In the following, after showing that the interplay of synaptic plasticity and scaling yields the described synaptic structure, we derive a population model of the neural system and analyze the underlying synaptic and neuronal dynamics to identify the required generic properties determining the synaptic adaptations. Finally, we demonstrate that our theoretical model matches and provides potential explanations for a series of experiments revealing the relation between neuronal dynamics and the allocation of memory (Yiu et al., 2014) and provide some experimentally verifiable predictions.

### 3.1. Formation and Allocation of the First Memory Representation

**Figure 2:**
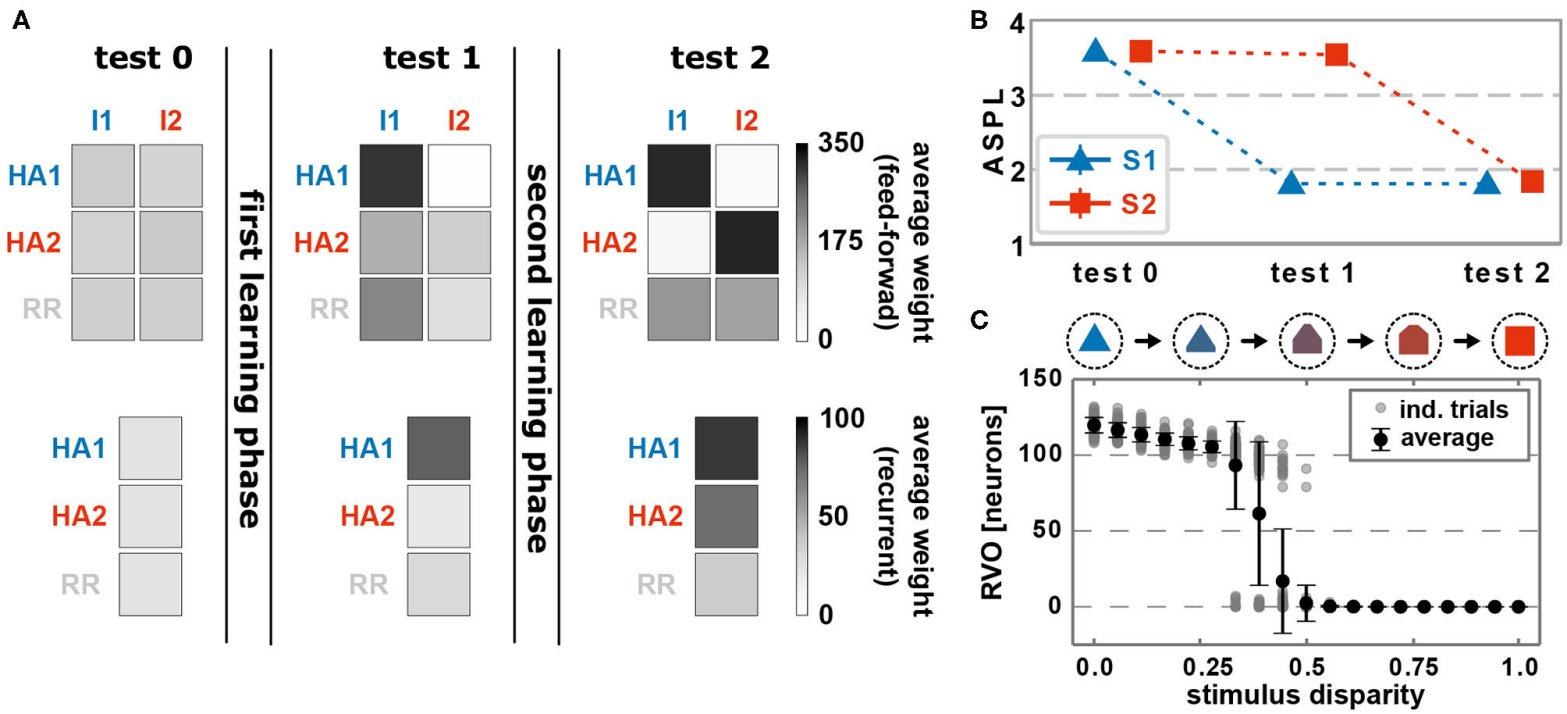
The interaction of conventional synaptic plasticity and scaling enables the stimulus-dependent formation and allocation of memory representations in a neuronal network model. **(A)** During the test phases, the resulting network structure is evaluated. Top row: average synaptic weight of feed-forward synapses from input populations I1 and I2 activated by the corresponding stimuli to the groups of neurons which become a HA (HA1 and HA2) and others (RR). Please note that we first train the network, then determine the resulting HAs with corresponding neurons, and retroactively sort the neurons into the HA-groups. Bottom row: average synaptic weight of recurrent synapses within the corresponding groups of neurons (HA1, HA2, and other neurons RR). Before learning (test 0), no specific synaptic structures are present. After the first learning phase (test 1), the first group of neurons becomes strongly interconnected, thus, a HA (HA1), which becomes also strongly connected to the active input population I1. (test 2) The second learning phase yields the formation of a second HA (HA2), which is linked to the second input population I2. **(B)** The formation of HAs is also indicated by the reduction of the average shortest path length (ASPL) between stimulus-activated neurons in the memory area (Error bars are small and overlapped by symbols). **(C)** After both learning phases, the response vector overlap (RVO) between neurons activated by S1 and activated by S2 depends non-linearly on the disparity between the stimulus patterns. **(A–C)** Data presented are mean values over 100 repetitions. Explicit values of mean and standard deviation are given in Supplementary Table S1.

Before learning, feed-forward as well as recurrent synapses, on average, do not show any structural bias (Figure 2A, test 0). The presentation of an environmental stimulus (e.g., S1 or S2) - via the activation of a stimulus-specific pattern within the input area (I1 or rather I2) - triggers the activation of a random pattern of active neurons in the memory area. We consider the average shortest path length (ASPL) between these neurons as a measure to evaluate to what degree these activated neurons are directly or indirectly connected with each other. Note that we assume a strongly interconnected local population of neurons as the basis for a HA (Hebb, 1949; Palm et al., 2014). In general, the ASPL is a graph theoretical measure to assess the average number of units (here neurons) along the shortest path between all pairs of units in a specific network or sub-network. Here, we consider only the pairs of highly activated neurons (details see Supplementary Material Section A) for two reasons: First, the considered synaptic processes of Hebbian plasticity and synaptic scaling depend on the neuronal activation such that the bulk of synaptic changes will happen at synapses connected to highly activated neurons. Second, given the bulk of synaptic changes we expect the formation of a HA to be correlated with highly activated neurons. Thus, if the ASPL-value equals one, all highly activated neurons are directly connected with each other. A high ASPL-value indicates that, on average, the most active neurons in the memory area are not directly connected with each other as given before learning (Figure 2B, test 0). Moreover, we can assume that the current activity pattern before learning is mainly determined by the random initial conditions in the feed-forward connections (number of connection as well as synaptic weight), since the recurrent connections were initialized homogeneusly. By contrast, if a stimulus (here stimulus S1) is repeatedly presented in a given time interval, the neuronal and synaptic dynamics of the network reshapes the pattern of activated neurons in the memory area such that the final pattern consists of a group of interconnected neurons (decrease in ASPL; Figure 2B, test 1). As shown in our previous studies (Tetzlaff et al., 2013; Nachstedt and Tetzlaff, 2017), the combination of synaptic plasticity and scaling together with a repeated activation of an interconnected group of neurons yields an average strengthening of the interconnecting recurrent synapses without significantly altering other synapses (Figure 2A, test 1, bottom; neurons are sorted into groups retroactively; see Supplementary Figure S2 for exemplary, complete weight matrices). Taken together with the decreased ASPL this indicates the stimulus-dependent formation of a HA during the first learning phase. However, do the self-organizing dynamics also link specifically the stimulus-neurons with the HA-neurons? A repeated presentation of stimulus S1 yields an on average strengthening of synapses projecting from the stimulus-neurons I1 to the whole memory area (Figure 2A, test 1, top). Essentially, the synapses from stimulus-I1-neurons to HA1-neurons have a significantly stronger increase in synaptic weights than the controls (HA2 and RR).

**Figure 3:**
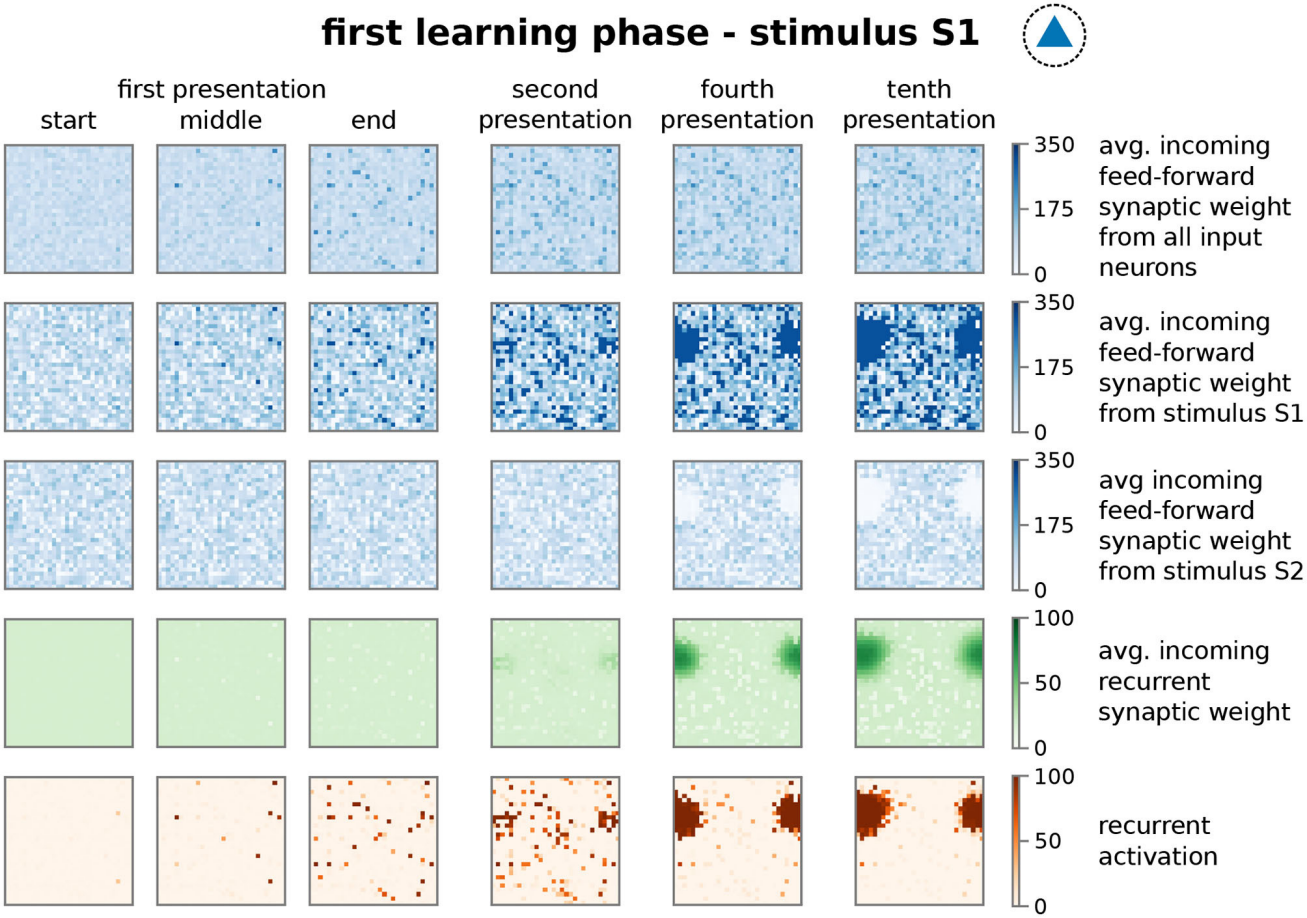
The repetitive presentation of a stimulus (here S1) triggers changes in feed-forward and recurrent synaptic weights as well as neural activities resulting to the proper formation and allocation of a HA. Each panel represents properties of the recurrent network in the memory area with neurons ordered on a 30 × 30 grid as indicated in Supplementary Figure S1A at different points of the protocol during the first learning phase. Thus, each dot in a panel represents a property of a neuron in the memory area, which is connected to the neighboring dots as shown in Supplementary Figure S1B. These properties are; first row: average feed-forward synaptic weights from all input neurons; second row: average feed-forward synaptic weights from the subset of I1-input neurons; third row: average feed-forward synaptic weights from the subset of I2-input neurons; forth row: average incoming synaptic weight from neurons of the memory area (recurrent synapses); fifth row: firing rate of the corresponding neuron. Please note that we consider torus-like periodic boundary conditions.

In more detail, presenting stimulus S1 initially activates an increasing number of mainly unconnected neurons in the memory area (Figure 3; first presentation, fifth row, dark red dots; nearby neurons are connected with each other as indicated in the inset of Figure 1B and Supplementary Figure S1B. This does not indicate the physical distance between neurons). However, the ongoing neuronal activation during following stimulus presentations increases the recurrent synaptic weights (Figure 3, fourth row) and also feed-forward weights from stimulus I1 to the neurons (second row), which, in turn, increases the activity. This positive feedback loop between synaptic weights and neuronal activity leads to the emergence of groups of activated neurons, which are directly connected with each other (second presentation, fifth row). Such an interconnected group grows out, incorporating more directly connected neurons, until inhibition limits its growth (see below) and, furthermore, suppresses sparse activation in the remaining neurons by competition (fourth to tenth presentation). The recurrent weights among HA-neurons are increased until an equilibrium between synaptic plasticity and scaling is reached (fourth row). Please note that, as our previous studies show (Tetzlaff et al., 2011, 2013), the interplay between these two mechanisms yields the existence of this equilibrium; otherwise synaptic weights would grow unbounded even if the neural activity is limited (see also detailed analysis below). Interestingly, the weights of the feed-forward synapses show a different dynamics as of the recurrent synapses. The average over all synaptic weights linking from the input area to each neuron in the memory area (first row) indicates that synapses connected to HA-neurons have a similar average weight compared to synapses connected to other neurons. This implies that the synaptic weight changes of the feed-forward connections to the HA-neurons are on average not significantly different than controls. However, if the feed-forward synapses are sorted according to the stimulus-affiliation of the pre-synaptic neuron, we see that only the weights of synapses from the S1-stimulus-neurons to the emerging HA-neurons are strengthened (second row, dark blue spot; see also Figure 2A, test 1, I1 to HA1), while weights of synapses from other stimulus-neurons to the HA-neurons are on average decreased (third row, white spot; see also Figure 2A, test 1, I2 to HA1). This implies a proper assignment of the stimulus to the newly formed HA during the learning phase resulting in a higher chance of activating the HA-neurons when the same stimulus is presented later again. Furthermore, a majority of these HA-neurons becomes active if a noisy version of the original stimulus is being presented during recall such that about 50% of the stimulus-I1-neurons have to be active to trigger the activation of about 80% of the HA-neurons (see Supplementary Figure S8). Such a “filling up” of active HA neurons by the strengthened recurrent network dynamics within the HA indicates the process of pattern completion (Hunsaker and Kesner, 2013). These results reveal that the interaction of synaptic plasticity and scaling self-organizes for a wide parameter regime (see Supplementary Figure S3) synaptic changes at recurrent and feed-forward connections to form and allocate a memory representation in a previously random neuronal network.

### 3.2. Formation and Allocation of a Second Memory Representation

After showing that the synaptic dynamics of the feed-forward connection does not impede the formation of a HA as internal representation of a stimulus, next, we will demonstrate that the recurrent dynamics (thus, a formed HA) does not obstruct the feed-forward dynamics given new stimuli. Clearly the presence of a memory representation can alter the self-organizing dynamics shown before, which could impede the proper formation and allocation of representations of further stimuli. For instance, the existence of a HA in the neuronal network could bias the adaptations of the feed-forward synapses such that a new stimulus is also assigned to this HA. This would imply that the neural circuit is unable to discriminate between the originally HA-associated stimulus and the new stimulus. Thus, to investigate the influence of prior learning, we repeatedly present a second, different stimulus S2 (second learning phase; Figure 1C) after the proper formation of the HA associated to stimulus S1 and analyse whether a second HA is formed, which is independent of the first one.

**Figure 4:**
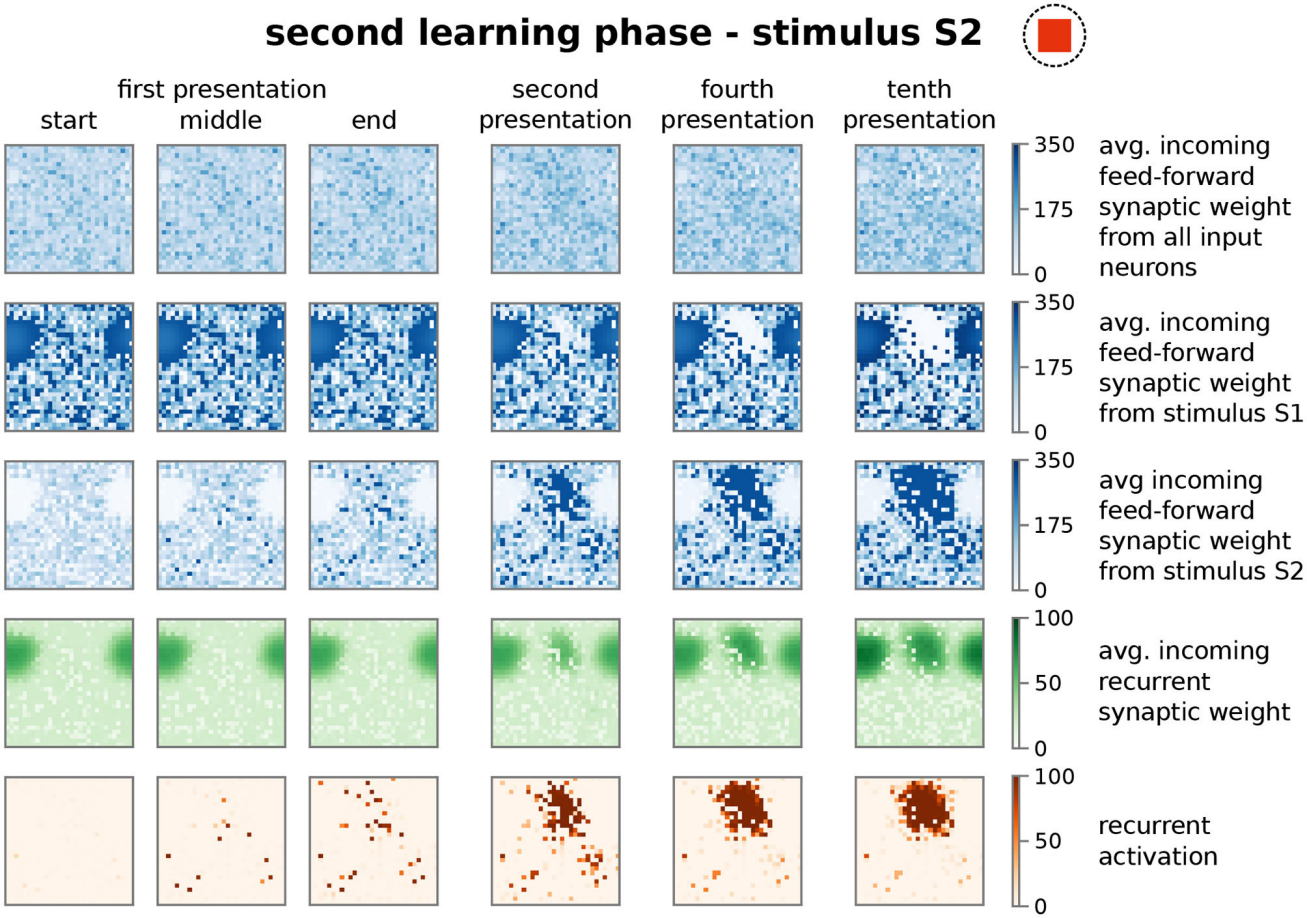
The presentation of a second stimulus (here S2) yields the formation of a second, distinct group of strongly interconnected neurons or HA. The structure of the sub-plots is the same as in Figure 3. The first learning phase yields the encoding of a highly interconnected sub-population of neurons in the memory area (Figures 2, 3). However, due to the interplay between synaptic plasticity and scaling this HA cannot be activated by the second stimulus S2. Instead, the process of initially scattered activation (dark red dots in the fifth row; first presentation) and the following neuronal and synaptic processes (fourth to tenth presentation), as described before, are repeated yielding the formation of a second HA representing stimulus S2. Please note that both representations do not overlap (see Supplementary Figure S2).

Similar to the first learning phase, the repeated presentation of stimulus S2 (here, stimulus-associated activity patterns in the input area I1 and I2 have a *stimulus disparity* equals 1 indicating no overlap between patterns; see Supplementary Material Section A) yields via activity pattern I2 in the input area the activation of a group of interconnected neurons in the memory area (decreased ASPL; Figure 2B, test 2, red). In addition, the stimulation triggers a strengthening of the corresponding recurrent synaptic weights (Figure 2A, bottom row, test 2, HA2; Figure 4). Thus, the stimulus-dependent formation of a new HA is not impeded by the existence of another HA. Furthermore, both HAs are distinguishable as they do not share any neuron in the memory area (Figure 4, forth row, tenth presentation; Supplementary Figure S2). As indicated by the response vector overlap (RVO, Figure 2C; basically the number of neurons activated by both stimuli), this depends on the disparity between stimuli; for quite dissimilar stimuli both HAs are separated (disparity ≳ 0.5 yields RVO ≈ 0), for more similar stimuli the system undergoes a state transition (0.3 ≲ disparity ≲ 0.5 yields RVO > 0), and for quite similar stimuli both stimuli activate basically the same group of neurons (disparity ≲ 0.3 yields RVO > 100 given that 120 ± 4 neurons are on average part of a HA; Supplementary Figure S4A). Note that the latter demonstrates that the network does correctly assign noisy versions of a learned stimulus pattern (*pattern completion* Hunsaker and Kesner, 2013) instead of forming a new HA, while the first case illustrates that the network performs *pattern separation* (Hunsaker and Kesner, 2013) to distinguish different stimuli. This indicates a correct assignment of the stimuli to the corresponding HAs, such that also in the presence of another HA the weight changes of synapses between input pattern and newly formed HA are adapted accordingly (Figure 2A, test 2, I2 to HA2).

Thus, the self-organizing dynamics yields the formation and allocation of a new HA during the second learning phase. Note that the synaptic weights of the initially encoded HA are not altered significantly during this phase (Figure 2A). But, although stimulus S1 is not present, the second learning phase leads to a weakening of synapses projecting from corresponding input neurons I1 to the newly formed HA considerably below control (Figure 2A, test 2, I2 to HA1). Similarly, during the first learning phase the synaptic weights between input neurons I2 and the first cell assembly are also weakened (Figure 2A, test 1, I2 to HA1). Apparently, this weakening of synapses from the other, non-assigned stimulus to a HA reduces the chance of spurious activations. In addition, as we will show in the following, this weakening is also an essential property enabling the proper functioning of the neural system. In summary, these results of our theoretical network model show that the self-organized processes resulting from the interplay between synaptic plasticity and scaling coordinates the synaptic and neuronal dynamics such that the proper formation and allocation of several memory representations without significant interferences between them is enabled.

### 3.3. Generic Properties of Synaptic Adaptations Required for the Formation and Allocation of Memory Representations

**Figure 5:**
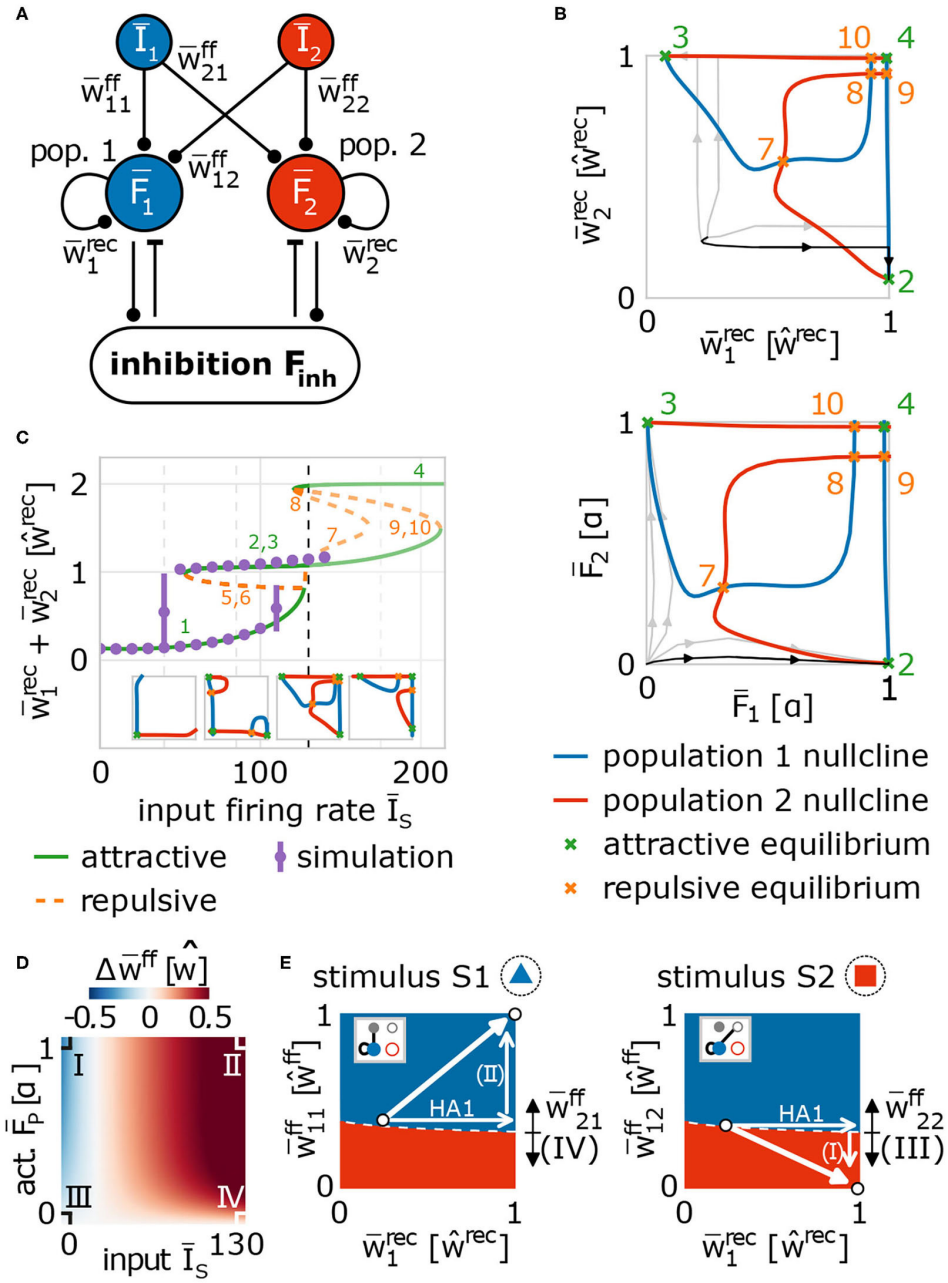
Population model of network dynamics enables analytical derivation of the underlying neuronal and synaptic dynamics. **(A)** Schema of the population model for averaged network dynamics (bars above variables indicate the average over all neurons in the population. Ī_*s*_: firing rate of input population *s* ∈ {1, 2}; ${\bar{F}}_{p}$: neural activity of population *p* ∈ {1, 2}; ${\bar{w}}_{ps}^{\text{ff}}$: weight of feed-forward synapses from population *s* to *p*; ${\bar{w}}_{p}^{\text{rec}}$: weight of recurrent synapses within population *p*. **(B)** The intersections of the population nullclines projected into weight (top) and activity (bottom) space reveal several fixed points (attractive: green; repulsive: orange) indicating the formation of a HA (green markers 2 and 3), if the system deviates from the identity line. Numbers correspond to labels of fixed points. **(C)** The bifurcation diagram (labels as in **(B)** and insets) of the network indicates that HAs are formed for a wide variety of input amplitudes (Ī_*A*_ ≳ 120). The dashed line illustrates the value used in **(B)**. Solutions of the full network model (purple dots) and population model match. **(D)** The dynamics of feed-forward synaptic weights depends on the firing rate of the input population and of the population in the memory area. There are four different cases (I–IV) determining the system dynamics. **(E)** These cases (indicated by arrows with Roman numbers: I–IV) together with the potentiation of recurrent synapses (arrow labeled HA1) yield the self-organized formation and allocation of HAs. Namely, during the first learning phase, synaptic changes drive the system (white dot) into regimes where either population 1 (blue) or population 2 (red) will represent the presented stimulus (left: stimulus S1; right: stimulus S2). Details see main text.

In order to obtain a more detailed understanding of the self-organizing coordination of synaptic and neuronal dynamics by synaptic plasticity and scaling underlying the reliable formation and allocation of memory representations, we have to reduce the complexity of the model to enable (partially) analytical investigations. As already indicated by the above shown results (Figure 2A), the main features of the self-organizing network dynamics can be described by considering the synaptic weights averaged over the given neuronal populations. Thus, we assume that the different involved neuronal populations in the input (input-pattern I1 and I2 neurons) and memory area (populations HA1 and HA2 becoming HAs in the memory area) are by themselves homogeneous allowing the derivation of a theoretical model describing the average population dynamics (Figure 5A). For this, we combine the neuronal dynamics of all neurons within such a population (groups of I1-, I2-, HA1-, HA2-neurons) and describe them by the average neuronal dynamics of the population such that we obtain four variables each describing the average firing rate of one population or group of neurons (I1: Ī_1_; I2: Ī_2_; HA1: ${\bar{F}}_{1}$; HA2: ${\bar{F}}_{2}$). Similarly, we combine the synaptic dynamics of all synapses to describe them by the average synaptic dynamics for all connections within a neuronal population (within HA1: ${\bar{w}}_{1}^{\text{rec}}$; within HA2: ${\bar{w}}_{2}^{\text{rec}}$) and for all connections between populations (from I1 to HA1: ${\bar{w}}_{11}^{\text{ff}}$; from I1 to HA2: ${\bar{w}}_{21}^{\text{ff}}$; from I2 to HA1: ${\bar{w}}_{12}^{\text{ff}}$; from I2 to HA2: ${\bar{w}}_{22}^{\text{ff}}$). As activities and weights of the remaining neurons and synapses (IR and RR groups of neurons in Figure 1D) remain small, in the following, we neglect their influence on the system dynamics. The inhibition is considered similarly (see section 2) and the values of some system parameters are taken from full network simulations (Supplementary Figure S4). Please note, we re-scale the average neuronal activities and the average synaptic weights such that, if the weights equal one, learning is completed (see section 2). By considering the average neuronal and synaptic dynamics, we map the main features of the complex network dynamics with ≈ *N*^2^ dimensions (*N* is the number of neurons in the network) to a 9-dimensional population model.

Given such a population model of our adaptive network, first, we investigate the formation of a memory representation in a blank, random neural circuit (thus, Ī_1_ > 0 and Ī_2_ = 0). As mentioned before, the advantage of using a population model is the reduced dimensionality enabling, amongst others, the analytical calculation of the nullclines of the system to classify its dynamics. Nullclines show states of the system in which the change in one dimension equals zero (Glendinning, 1994; Izhikevich, 2007). Thus, the intersection points of all nullclines of a system are the fixed points of the systems dynamics describing the system states in which no change occurs. Such fixed points can either be stable (attractive), thus the system will tend to converge into this point or state, or unstable (repulsive) meaning that the system will move away from this state (Supplementary Material Section C). Such dynamics can be summarized in a phase space diagram, in which each point indicates a possible state of the system and we can determine from the relative position of this state to the nullclines and fixed points to which new state the system will go to. The population model of the network model has 9 dimensions, thus, 9 nullclines and a 9-dimensional phase space (Figure 5A); however, by inserting solutions of some nullclines into others (Supplementary Material Section B), we can project the dynamics onto two dimensions for visualization. In the following, we project the dynamics onto the average recurrent synaptic weights of both populations in the memory area (${\bar{w}}_{1}^{\text{rec}}$, ${\bar{w}}_{2}^{\text{rec}}$) to investigate the dynamics underlying the formation of a HA during the first learning phase (Figure 5B, top; please see Figure 5B, bottom, for corresponding activity levels).

Thus, the projection of the solutions of the nullclines of the system dynamics onto the average recurrent synaptic weights of the two neuronal groups shows that the recurrent dynamics during the first learning phase are dominated by three fixed points: one is unstable (orange, 7; more specifically, it is a saddle point) and two are stable (green, 2 and 3). As the recurrent synaptic weights before learning are in general rather weak (which “sets” the initial state in this diagram), the fixed points 4, 8, 9, 10 cannot be reached by the system and, thus, they do not influence the here discussed dynamics. The two stable fixed points represent that one of the two neuronal populations becomes a strongly interconnected HA, while the other remains in a weakly interconnected state. For instance, in state 2 the first population is a HA as it is strongly interconnected (${\bar{w}}_{1}^{\text{rec}}\gg 0$) and the second population of neurons remains weakly connected (${\bar{w}}_{2}^{\text{rec}}$ has only about 10% of the value of ${\bar{w}}_{1}^{\text{rec}}$). The unstable or repulsive fixed point lies on the identity line (${\bar{w}}_{1}^{\text{rec}}={\bar{w}}_{2}^{\text{rec}}$) having the same distance to both stable, attractive states. The resulting mirror symmetry in the phase space implies that the dynamics on the one side of the identity line, reaching the stable fixed point lying on this side, equals the dynamics on the other side. Please note that the form of the nullclines and, thus, the existence and positions of the fixed points of the system dynamics depend on the mechanisms determining the synaptic adaptations. In other words, given a strong stimulus, the interplay between synaptic plasticity and scaling coordinates the recurrent synaptic dynamics such that the system by itself has to form a HA (reach one of both stable states). The question, which of both stable states is reached, translates into the question, to which group of neurons will the stimulus be allocated. As both groups are before learning quite similar, the initial state of the system will be close to the identity line. Only a minor difference between both groups (e.g., slightly different recurrent synaptic weights or a little different number of feed-forward synapses) results to a small variation in the initial condition of the first learning phase such that the system is slightly off the identity line (see traces for examples). Given the difference, the system will be on one side of the identity line and converge to the corresponding fixed point implying that the corresponding group of neurons will become the internal representation (e.g., black trace). Note that this symmetry-dependent formation of a HA is quite robust as long as the input firing rate is above a certain threshold (Ī_*A*_ ≳ 120), which agrees with results from the more detailed network model discussed before (purple dots; Figure 5C). Below this threshold, the system remains in a state both groups of neurons are not becoming HAs (state 1 in Figure 5C; see also first two insets). Thus, the existence of the threshold predicts that a new, to-be-learned stimulus has to be able to evoke sufficient activity in the input area (above the threshold) to trigger the processes of memory formation; otherwise, the system will not learn.

In parallel to the development of the recurrent synaptic weights, the synaptic weights of the feed-forward connections change to assure proper memory allocation. Thus, we derive analytically the activity-dependency of the interaction of synaptic plasticity and scaling and obtain the change of the feed-forward synaptic weights ($\Delta {\bar{w}}^{\text{ff}}$) being expected during the first learning phase, given different activity conditions of the input (Ī_*s*_) and HA-populations (${\bar{F}}_{p}$, *s, p* ∈ {1, 2}; Figure 5D and Supplementary Material Section D). As expected, the combination of both activity levels for a certain duration determines whether the weights of the feed-forward synapses are potentiated (red), depressed (blue), or not significantly adapted (white). In general, if both activities are on a quite high level, synapses are potentiated (case II; so-called homosynaptic potentiation; Miller, 1996). If the pre-synaptic activity (input population) is on a low level and the post-synaptic activity (HA-population) is on a high level, on average, feed-forward synapses are depressed (case I; so-called heterosynaptic depression; Miller, 1996). However, if the post-synaptic activity is low, synaptic changes are negligible regardless of the level of pre-synaptic activity (cases III and IV).

The different parts of recurrent and feed-forward synaptic dynamics together lead to the formation and allocation of a HA as described in the following. For this, given the presentation of a to-be-learned stimulus, we have to consider the basins of attraction of the system in the phase space (Figure 5E) projected onto different types of connections (insets; gray indicates the active input population). If the system is in a certain state, we marked by the color of this state which group of neurons will become a HA and will be assigned to the stimulus presented (left: S1 is presented; right: S2 is presented). The mirror symmetry described before (Figure 5B) maps to the boundary (white dashed line in Figure 5E) between both basins of attraction (blue: population 1 becomes the internal representation; red: population 2 becomes the HA). Thus, during the first learning phase (stimulus S1; Figure 5E, left), a small variation in initial conditions breaks the symmetry such that the system is, in the example highlighted in Figure 5B (black trace), in the basin of attraction of population 1 becoming the internal representation (dot nearby symmetry line in blue area). This leads to the strengthening of the recurrent synapses within population 1 forming a HA (increase of ${\bar{w}}_{1}^{\text{rec}}$; Figures 5B,E). In parallel, the synaptic strengthening induces an increase of the activity level of the population (${\bar{F}}_{1}$; black trace in Figure 5B, bottom) yielding, together with the high activity level of input population I1 (Ī_1_ ≫ 0), an average increase of the corresponding feed-forward synapses (${\bar{w}}_{11}^{\text{ff}}$; case II in Figure 5D). These synaptic changes push the system further away from the symmetry condition (white arrows; Figure 5E, left) implying a more stable memory representation. Note that changing the strength of synapses connecting input population I1 with population 2 (${\bar{w}}_{21}^{\text{ff}}$) could result in a shift of the symmetry condition (indicated by black arrows) counteracting the stabilization process. However, this effect is circumvented by the system, as the second population has a low activity level and, therefore, corresponding feed-forward synapses are not adapted (case IV in Figure 5D). Thus, during the first learning phase, the formation and allocation of an internal representation is dictated by the subdivision of the system phase space into different basins of attraction of the stable fixed points such that small variations in the before-learning state of the network predetermines the dynamics during learning. This subdivision, in turn, emerges from the interplay of synaptic plasticity and scaling.

**Figure 6:**
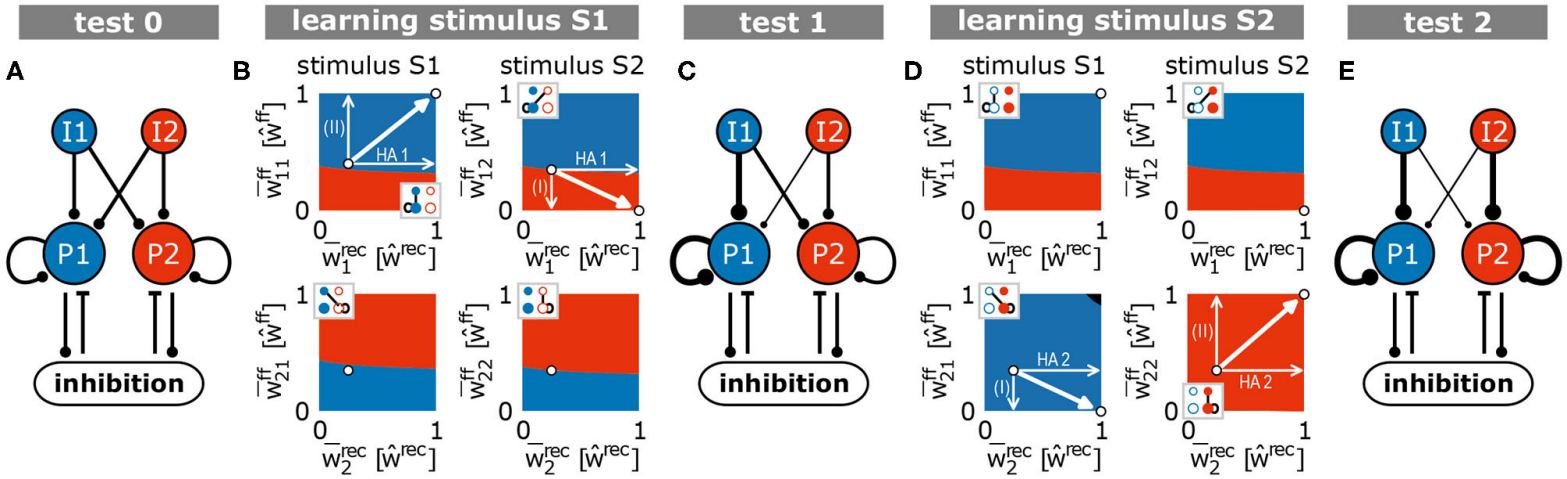
Summary of the synaptic changes and their implication on the formation and allocation of memory representations. The interaction of synaptic plasticity and scaling brings a blank network [**(A)** test 0] during the first learning phase **(B)** to an intermediate state [**(C)** test 1]. From this intermediate state, the second learning phase **(D)** leads the system to the desired end state [**(E)** test 2], in which each stimulus is allocated to one HA (*S*1/*I*1 to pop. 1/HA1 and *S*2/*I*2 to pop. 2/HA2). **(A,C,E)** Thickness of lines is proportional to average synaptic weight. **(B,D)** Similar to panels in Figure 5E. Black area indicates regimes in which both populations would be assigned to the corresponding stimulus.

How do these synaptic and neuronal dynamics of the allocation and formation of the first HA influence the dynamics of the second learning phase? In general, the formation of a HA acts as a variation or perturbation of the initial condition breaking the symmetry for the second learning phase (stimulus S2; Figure 5E, right). The formation of the first HA pushes the system into the blue area (HA1-arrow). This indicates that, if stimulus S2 is presented, the feed-forward synapses would be adapted such that population 1 would also represent stimulus S2. This would impede the discrimination ability of the network between stimulus S1 and S2. However, during the first learning phase, as the input population I2 of stimulus S2 is inactive, synapses projecting from I2-input neurons to population 1 neurons are depressed (case I in Figure 5D; downward arrow in Figure 5E, right) and the system switches into the red area. This area indicates that, if stimulus S2 is presented during the second learning phase, population 2 would form a HA representing stimulus S2 and not population 1. Please note that this switch can be impeded by adapting the connections from the I2-input population to population 2 during the first learning phase (${\bar{w}}_{22}^{\text{ff}}$) shifting the symmetry condition (black arrows in Figure 5E, right). But, similar to before, this effect is circumvented by the system, as population 2 is basically inactive resulting to case III (Figure 5D). Thus, after the first learning phase, the synaptic dynamics regulated by the combination of synaptic plasticity and scaling drives the system into an intermediate state, which implies that the system will definitely form a new HA during the second learning phase (see Figure 6 for further phase space projections and dynamics during second learning phase). These results indicate that this intermediate state can only be reached if synaptic adaptations comprise three properties implied by the four cases I-IV (Figure 5D): (i) homosynaptic potentiation (case I), (ii) heterosynaptic depression (case II), and (iii) the down-regulation of synaptic weight changes by the post-synaptic activity level (cases III and IV).

### 3.4. Modeling Experimental Findings of Competition-Based Memory Allocation

**Figure 7:**
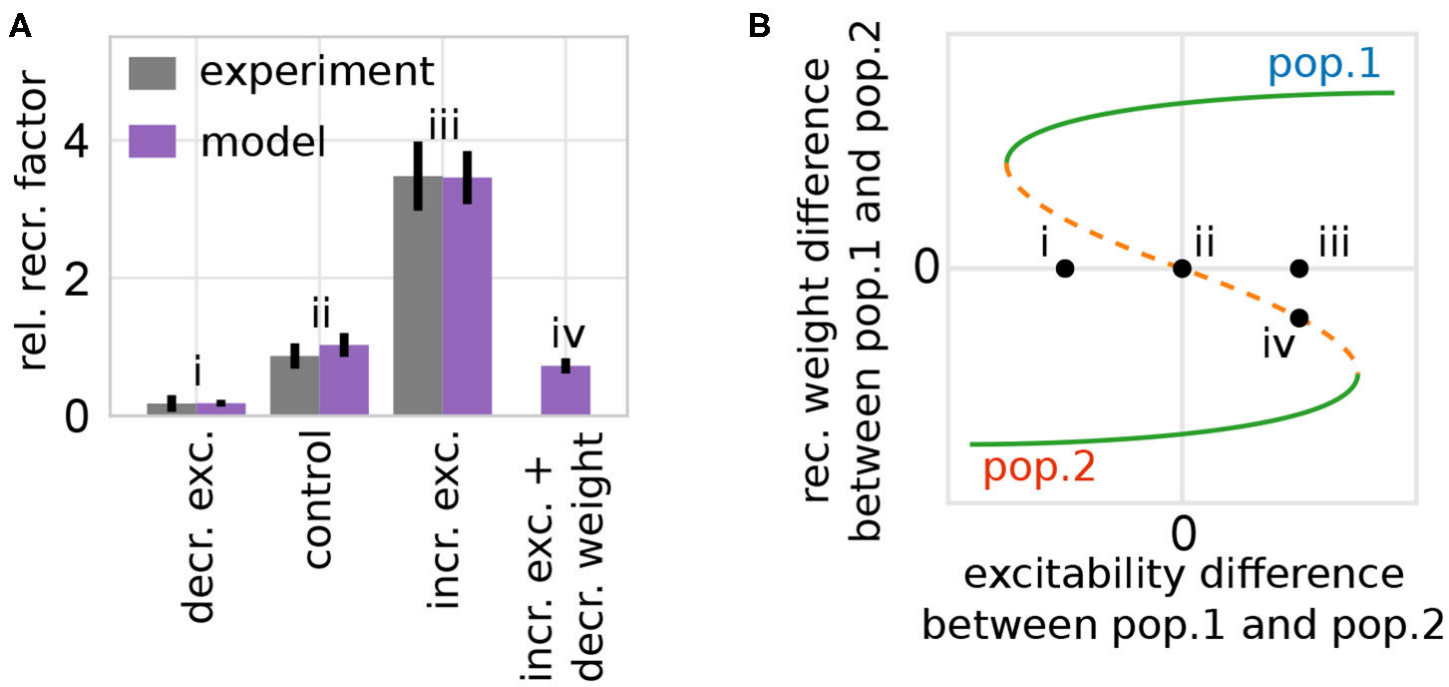
The model of synaptic plasticity and scaling matches experimental *in-vivo* data and provides experimentally verifiable predictions. **(A)** The artificial modification of the excitability of a subset of neurons alters the probability of these neurons to become part of a memory representation (normalized to control). Experimental data are taken from Yiu et al. (2014). Data presented are mean values with standard error of the mean. Labels correspond to instances shown in **(B)**. **(B)** The alteration of the excitability of one group of neurons (here pop. 1) compared to others yields a shift of the positions of the system's fixed points and basins of attractions (here shown schematically; for details see Supplementary Figure S5) inducing a bias toward one population (e.g., from instance ii to iii by increasing the excitability of population 1). **(A,B)** The model analysis yields the prediction that this excitability-induced bias can be counterbalanced by, for instance, additionally decreasing the average synaptic weight of the manipulated population before learning (here by a factor of 0.1). This additional manipulation shifts the initial state of the network back to the symmetry condition (orange; instance iv).

In addition to the described three properties, our model implies the existence of a symmetry condition underlying the formation and allocation of memory representations. Small variations in the initial condition of the system suffice to break this symmetry. These variations could be, aside from noise, enforced experimentally by adapting neuronal parameters in a local group of neurons. One experimental study (Yiu et al., 2014) indicates that the probability of a group of neurons to become part of a newly formed memory representation can be influenced by changing their excitability genetically (e.g., by varying the CREB concentration). We reproduced such manipulations based on experiments investigating fear memory. Thus, the stimulus considered here in our model is related to pain. Please note that the detailed learning protocol in experiment and model are different; however, detailed biological models (Kim et al., 2013a,b) indicate that fear learning also leads to the formation of Hebbian cell assembly like memory representations, as in our model during learning. Thus, similar to the experimental procedures, we adapt the neuronal excitability of a group of neurons and analyze the resulting data accordingly (see section 2). Thus, we determined the probability of a single neuron to become part of a HA averaged over the whole manipulated group of neurons (relative recruitment factor) and compared the results to experimental findings (Figure 7A).

On the one hand, if the excitability of a group of neurons is artificially increased briefly before learning, the probability of these neurons to become part of the memory representation is significantly enhanced. On the other hand, if the excitability is decreased, the neurons are less likely to become part of the representation. Considering the theoretical results shown before (Figure 5B), this phenomenon can be explained as follows: the manipulation of the excitability in one population of neurons changes the distance between the repulsive state (orange; Figure 7B) to the two attractive states (green). Thus, an increased (decreased) excitability yields a larger (smaller) distance between the repulsive state and the attractive state related to the manipulated population (e.g., Figure 7B instance iii for increased excitability in population 1). This larger (smaller) distance implies a changed basin of attraction of the manipulated population enhancing the chance that the initial condition of the network (black dots) lies within this basin. This implies an increase (decrease) of the probability that this group of neurons becomes a HA, as depicted by the variation of the experimentally measured single neuron probability. In other words, the increased excitability leads on average to an easier and stronger activation of this neuronal group, which in turn changes the balance between Hebbian synaptic plasticity and synaptic scaling, such that the synapses of this group are potentiated faster yielding a stronger activation and so on. In addition, the competing neuronal groups are repressed via mutual inhibition further increasing the probability of neurons with enhanced excitability to become part of the memory representation.

This theoretical analysis yields the prediction that the measured effects will be altered by manipulating other parameters. For instance, if the synaptic weight of the population with increased excitability is on average decreased before stimulus presentation (e.g., by PORCN; Erlenhardt et al., 2016), the network's initial condition is shifted such that the CREB-induced influence on the relative recruitment factor is counterbalanced (Figure 7A, instance iv). Please note that the general approach of our model indicates that this prediction could be verified in diverse learning paradigms.

## 4. Discussion

Our theoretical study indicates that the formation as well as the allocation of memory representations in neuronal networks depend on the self-organized coordination of synaptic changes at feed-forward and recurrent synapses. By deriving a population model, we provide the first theoretical analysis identifying a symmetry mechanism underlying the problem of memory allocation. By this model, we also obtain the dependencies of the mechanism on network and neuron parameters. Furthermore, we predict that the combined dynamics of synaptic plasticity and scaling could be sufficient for yielding the self-organized coordination as it implies three generic properties: (i) homosynaptic potentiation, (ii) heterosynaptic depression, and (iii) the down-regulation of synaptic weight changes by the post-synaptic activity level.

Homosynaptic potentiation is a well-known concept being directly related to the dynamics of long-term potentiation (Bliss and Lomo, 1973; Levy and Steward, 1983; Bi and Poo, 1998; Dayan and Abbott, 2001; Gerstner and Kistler, 2002; Malenka and Bear, 2004; Feldman, 2009). Heterosynaptic depression implies the competition between different synapses or inputs that are connected to the same postsynaptic neuron (Miller and MacKay, 1994; Miller, 1996). Several theoretical studies indicate that competition can be implemented by different homeostatic mechanisms such as intrinsic plasticity, weight normalization, or synaptic scaling (Bienenstock et al., 1982; Abraham and Bear, 1996; Yeung et al., 2004; Keck et al., 2012; Yger and Gilson, 2015; Miner and Triesch, 2016; Triesch et al., 2018; Kruppel and Tetzlaff, 2020) each based on diverse biological principles (Turrigiano et al., 1998; Zhang and Linden, 2003; Turrigiano and Nelson, 2004; Triesch et al., 2018).

In this study we considered synaptic scaling that is a homeostatic mechanism found in several brain areas and under various experimental condition (Turrigiano et al., 1998; Burrone et al., 2002; Hengen et al., 2013; Keck et al., 2013). In principle this mechanism detects deviations of the postsynaptic activity from a desired target value (Turrigiano and Nelson, 2004; Ibata et al., 2008); thus, if the activity is larger than the target value, the synaptic weights are decreased and vice versa (see second summand in Equations 5 and 6). On the one hand, the detection of the deviation has to act on a time scale of seconds to several minutes (Zenke et al., 2013) such that it could be implemented by variations of calcium concentrations (Turrigiano, 2008, 2011). In this study we neglected these calcium dynamics and considered an immediate detection of the activity deviation ($ẇ\propto F^{\text{T}}-F_{\text{post}}$). On the other hand, synaptic scaling influences the synaptic dynamics usually on a slower time scale than long-term synaptic plasticity (but see also Bourne and Harris, 2011), which we implement in our model by considering κ >> 1. Despite this slow time scale, theoretical and numerical analyses indicate that synaptic scaling keeps the synaptic dynamics within a healthy, functional regime (Tetzlaff et al., 2011, 2013, 2015; Toyoizumi et al., 2014; Yger and Gilson, 2015). Furthermore, the combination of long-term potentiation and homeostatic plasticity implies the two properties of homosynaptic potentiation and heterosynaptic depression and this combination is required in recurrent neuronal networks to dynamically form memory representations (Tetzlaff et al., 2013; Litwin-Kumar and Doiron, 2014; Zenke et al., 2015). However, these studies do not consider the feed-forward synaptic dynamics. Studies analyzing feed-forward dynamics, such as the self-organization of cortical maps (Kohonen, 1982; Sullivan and de Sa, 2006; Stevens et al., 2013), also indicate the importance of homosynaptic potentiation and heterosynaptic depression. However, these studies do not consider the recurrent synaptic dynamics. Only by considering both feed-forward *and* recurrent synaptic dynamics, we revealed the requirement of property (iii) that a low level of post-synaptic activity curtails the synaptic changes which is also supported by experimental evidence (Sjostrom et al., 2001; Graupner and Brunel, 2010). Note that property (iii) is realized by both mechanisms: Hebbian synaptic plasticity as well as synaptic scaling. By contrast, property (i) is implemented by Hebbian synaptic plasticity only and property (ii) is realized by synaptic scaling only. This indicates that synaptic scaling could have an essential role in the allocation and formation of multiple memory representations beyond the widely assumed stabilization of neural network dynamics (Abbott and Nelson, 2000; Turrigiano and Nelson, 2004; Tetzlaff et al., 2011; Turrigiano, 2017; Zenke and Gerstner, 2017). So far, to the best of our knowledge, experimental studies did not survey synaptic scaling in the context of memory. Thus, new experimental setups are required to investigate whether synaptic scaling influences the dynamics of memory and to verify its role as predicted by our model. Please note that other adaptive mechanisms or combinations of these (e.g., intrinsic plasticity, Zhang and Linden, 2003; Triesch, 2007, structural plasticity, Fauth and Tetzlaff, 2016; Gallinaro and Rotter, 2018, voltage-based synaptic plasticity, Clopath et al., 2010) could also implement all three properties (e.g., the BCM-rule does not implement all three properties and does not seem to yield the desired dynamics; see Supplementary Figures S9, S10). The identification of such combinations requires further investigations. However, as indicated by our results, the properties of synaptic plasticity and scaling could be sufficient such that the combination of both could lead to the desired self-organized coordination of synaptic changes at feed-forward and recurrent synapses.

Similar to previous studies (Tetzlaff et al., 2013, 2015; Nachstedt and Tetzlaff, 2017), we consider here an abstract model to describe the neuronal and synaptic dynamics of the network. Despite the abstract level of description, the model matches experimental *in-vivo* data of memory allocation. Other theoretical models match similar experimental data (Kim et al., 2013a; Kastellakis et al., 2016); however, these models are of greater biological detail including more dynamic processes (e.g., short-term plasticity). However, only by considering an abstract model, we have been able to derive analytical expressions such that we could find the underlying nullclines and the requirement of the three generic properties yielding the proper formation and allocation of memories. Remarkably, the synaptic plasticity processes considered in the detailed models (Kim et al., 2013a,b; Kastellakis et al., 2016) also imply the three generic properties (i-iii) supporting our findings. Further investigations are required to assess possible differences between different realizations of the three generic properties. For this, the here used theoretical methods from the field of non-linear dynamics (Glendinning, 1994; Izhikevich, 2007) seem to be promising given their ability to derive and classify fundamental system dynamics, which can be verified by experiments.

In our model the neuronal and synaptic dynamics always yields the formation of separated memory representations for different stimuli. Hereby, Figure 2C and Supplementary Figure S8 together indicate that a new memory representation is formed when the new stimulus does not trigger the activation of a stored representation. Thus, the non-activation of any memory representation by a stimulus could act as an internal novelty signal triggering learning. However, a new stimulus being partially similar to a learned one could lead to interferences, as in the current model no overlaps that consists of neurons encoding more than one stimulus at the same time are being formed. In particular, the process of heterosynaptic depression inherently impedes the formation of an overlap between memory representations (Tetzlaff et al., 2013). Any two HAs sharing a sub-population of neurons - and thus sharing strong interconnections - would be separated once either of the two turns active: high activation of HA1 would suppress HA2 and thus synapses from HA2 to HA1 are depressed (and vice versa). This mechanism necessarily affects the capacity of the circuit. A preliminary test shows that within our set of parameters HAs start to interfere when memorizing a fifth HA (see Supplementary Material Section F). The interference is expressed in a way that several neurons, which belonged to a different HA, are re-assigned to the newly formed one and that they do not tend to encode several stimuli (no overlap). This implies that the number of HAs that can be stored in the circuit depends on the number of neurons or the size of a HA by optimally “packing” the HAs into the network without overlaps. If a large number of neurons is already part of a HA, a new learning stimulus would lead to a kind of catastrophic forgetting. However, in order to reach a more thorough understanding of these results, expansive additional investigations are required considering methods to evaluate the relations between HAs systematically (Babadi and Sompolinsky, 2014; Kruppel and Tetzlaff, 2020).

Experimental results indicate that memory representations can overlap (Cai et al., 2016; Holtmaat and Caroni, 2016; Yokose et al., 2017) and, in addition, theoretical studies show that overlaps increase the storage capacity of a neuronal network (Tsodyks and Feigelman, 1988) and can support memory recall (Recanatesi et al., 2015). To partially counterbalance the effect of heterosynaptic depression to allow the formation of overlaps, further time-dependent processes are required. For instance, the CREB-induced enhancement of neuronal excitability biases the neuronal and synaptic dynamics such that the respective subgroup of neurons is more likely to be involved in the formation of a memory representation (Figure 7; Kim et al., 2013a; Yiu et al., 2014). Furthermore, the dynamics of CREB seem to be time-dependent (Yiu et al., 2014; Frankland and Josselyn, 2015; Kastellakis et al., 2016). Therefore, the enhancement of CREB can counterbalance heterosynaptic depression for a given period of time and, by this, could enable the formation of overlaps. We expect that the impact of such time-dependent processes on the dynamics of memories can be integrated into the here-proposed model to analyse the detailed formation of overlaps between memory representations. Thus, our study shows that the interplay between synaptic plasticity and scaling is required to include all three generic properties of synaptic adaptation enabling a proper formation and allocation of memories. In addition, given the here-derived theoretical model, other mechanisms can be included to investigate systematically their functional implication on the self-organized, complex system dynamics underlying the multitude of memory processes.

## Data Availability Statement

The datasets generated for this study are available on request to the corresponding author.

## Author Contributions

JA contributed the network simulations. TN contributed the population model. CT designed and supervised the study. All authors reviewed the manuscript.

## Conflict of Interest

The authors declare that the research was conducted in the absence of any commercial or financial relationships that could be construed as a potential conflict of interest.
