# Supplementary material for: The Interplay of Synaptic Plasticity and Scaling Enables Self-Organized Formation and Allocation of Multiple Memory Representations
Source: Front Neural Circuits. 2020 Oct 7;14:541728. doi: 10.3389/fncir.2020.541728 (PMC7575689; doi:10.3389/fncir.2020.541728)
Supplement: Supplementary file 1 [file Data_Sheet_1.PDF]

Supplementary Material

SECTION A: MEASURES USED IN THE NETWORK SIMULATION

Average shortest path length

The *average shortest path length* (ASPL, Figure 2 B) is considered here as a measure to analyze the spatial distribution of activation within the memory area. A high ASPL between neurons indicates that these neurons are spatially broadly distributed across the memory area. By contrast, a low ASPL indicates that the neurons are clustered. In particular, as strongly activated neurons are supposed to become part of a memory representation, we focus on the distribution of highly activated neurons. For this, for each trial, we identified the 10% of neurons with the highest activity level (index set P) and calculated the shortest path length (SPL; Rubinov and Sporns, 2010); using the *networkX* package for *Python*) between them and averaged over all those paths (denoted by $\langle \cdot \rangle$):

$$\mathrm{ASPL} = \langle \mathrm{SPL}_{\mathrm{i,j}} \rangle_{i,j \in P, \mathrm{i} \neq \mathrm{j}}. \tag{S1}$$

Dynamic equilibria of synaptic weights

The *average outgoing recurrent synaptic weight* (Figure 2 A) is a measure of the interconnection within a neuronal sub-population in the memory area (index set Q). We therefore averaged the synaptic weight over all the connections among neurons within the sup-population:

$$\bar{w}^{\mathrm{rec}} = \langle w_{\mathrm{i,j}}^{\mathrm{rec}} \rangle_{i,j \in Q, \mathrm{i} \neq \mathrm{j}}. \tag{S2}$$

The *average incoming feed-forward synaptic weight* (Figure 2 F,G) is the average synaptic weight of connections between a sub-population in the memory area (index set Q) and a specific stimulus pattern in the input area (index set H):

$$\bar{w}^{\mathrm{ff}} = \langle w_{\mathrm{i,k}}^{\mathrm{ff}} \rangle_{i \in Q, k \in H}. \tag{S3}$$

Response disparity dependent on stimulus similarity

To analyse the response disparity, stimulus S1 is presented 10 times for 5 sec with 1 sec pause in between to form a single HA. After that, plasticity is shut off and we present variations of stimulus S1 with increasing *stimulus disparity* until the stimulus equals stimulus S2 (Figure 2 C). Stimulus disparity measures the relative amount of non-overlap between two stimulus patterns - in this case stimulus S1 and its variation (in the following called stimulus S1'). Both stimuli are of identical size $N^{\mathrm{S}} = 0.5 \cdot N^{\mathrm{I}}$, so that the stimulus disparity is calculated as follows:

$$\textit{stimulus disparity}\left(S1,\, S1'\right) = 1 - \frac{1}{N^{\mathrm{S}}} \cdot \sum_{\mathrm{k}}^{N^{\mathrm{I}}} S_{\mathrm{k}}(S1) \cdot S_{\mathrm{k}}(S1'), \tag{S4}$$

with binary stimulus patterns for a given stimulus $X \in S1, S1'$:

$$S_{\mathrm{k}}(X) = \begin{cases} 1, & \text{if } I_{\mathrm{k}}(X) = 130, \\ 0, & \text{if } I_{\mathrm{k}}(X) = 0. \end{cases} \tag{S5}$$

Thus, a stimulus disparity equal zero describes two identical stimuli, whereas a disparity equal one indicates two non-overlapping stimulus patterns. The input area size of $N^{\mathrm{I}} = 36$ allows for 18 steps in variation of $5.\bar{5}\%$ each. At the end of each presentation, we compare the resulting response in the memory area with the one at the end of the learning phase (i.e. the response to the original stimulus S1). The *response vector overlap* (RVO; Figure 2 C) describes the similarity between the response patterns in the memory area due to the presentation of stimuli S1 and S1':

$$RVO(S1,\, S1') \;=\; \sum_{\mathrm{i}}^{\mathrm{N^M}} R_{\mathrm{i}}(S1) \cdot R_{\mathrm{i}}(S1') \tag{S6}$$

with binary response of neuron i to a given stimulus $X \in S1, S1'$:

$$R_{\mathrm{i}}(X) = \begin{cases} 1, & \text{if } F_{\mathrm{i}}(X) \geqslant 0.5 \cdot \alpha, \\ 0, & \text{else.} \end{cases} \tag{S7}$$

Explicit network simulation results

Table S1. Explicit results depicted in Figure 2 A,B: average shortest path length (ASPL) and average synaptic weights (mean±std).

	**test0**	**test1**	**test2**
$\bar{w}^{\mathrm{ff}}_{11}$	107 ± 36	290 ± 11	302 ± 11
$\bar{w}^{\mathrm{ff}}_{12}$	96 ± 22	145 ± 19	25 ± 15
$\bar{w}^{\mathrm{ff}}_{21}$	95 ± 21	3.0 ± 0.1	15.0 ± 0.1
$\bar{w}^{\mathrm{ff}}_{22}$	108 ± 36	103 ± 36	305.0 ± 0.0
$\bar{w}^{\mathrm{rec}}_{1}$	19.4 ± 0.0	69 ± 10	81 ± 22
$\bar{w}^{\mathrm{rec}}_{2}$	19.4 ± 0.0	17 ± 5	63 ± 20
$\bar{w}^{\mathrm{rec}}_{\mathrm{RR}}$	19.4 ± 0.0	25 ± 6	30 ± 11
ASPL S1	3.59 ± 0.02	1.81 ± 0.01	1.81 ± 0.02
ASPL S2	3.59 ± 0.02	3.55 ± 0.04	1.84 ± 0.04

SECTION B: NULLCLINES AND EQUILIBRIA

The equilibrium values $\bar{w}_i^{\mathrm{ff},*}$ and $\bar{w}_i^{\mathrm{rec},*}$ of the feed-forward and recurrent weights can be obtained as a function of the equilibrium values of the population activities $\bar{F}_i^*$ from Equation 10 and Equation 11:

$$\bar{w}_{ik}^{\mathrm{ff},*}(\bar{F}_i^*) = \sqrt{\frac{\kappa^{\mathrm{ff}} \bar{F}_i^* \bar{I}_k}{\bar{F}_i^* - F^{\mathrm{T}}}}. \tag{S8}$$

$$\bar{w}_i^{\mathrm{rec},*}(\bar{F}_i^*) = \sqrt{\frac{\kappa^{\mathrm{rec}} (\bar{F}_i^*)^2}{\bar{F}_i^* - F^{\mathrm{T}}}}, \tag{S9}$$

The equilibrium value $\bar{u}^*_{\text{inh}}$ of the membrane potential of the inhibitory population can be formulated as a function of $\bar{F}^*_1$ and $\bar{F}^*_2$ based on Equation 8 (here, $N_1 = N_2 = N$):

$$\bar{u}^*_{\text{inh}}(\bar{F}^*_1, \bar{F}^*_2) = R_{\text{inh}}\tau_{\text{inh}}(w_{\text{inh},1}N_1\bar{F}^*_1 + w_{\text{inh},2}N_2\bar{F}^*_2). \tag{S10}$$

By inserting Equations S9, S8 and S10 into Equation 7 and using $\bar{F}^*_i = F(\bar{u}^*_i)$, we obtain a system of the two population nullclines that only depends on the equilibrium values $\bar{u}^*_1$ and $\bar{u}^*_2$ ($i \in \{1, 2\}$):

$$0 = -\frac{\bar{u}^*_i}{\tau} + R\left(\bar{n}^{\text{rec}}_i\bar{w}^{\text{rec},*}_i(\bar{F}^*_i)\bar{F}^*_i + w_{i,\text{inh}}\bar{F}^*_{\text{inh}}(\bar{F}^*_1, \bar{F}^*_2) + \sum_k \bar{n}^{\text{ff}}\bar{w}^{\text{ff},*}_{ik}(\bar{F}^*_i)\bar{I}_k\right). \tag{S11}$$

We solve this system numerically to receive the equilibrium values $\bar{u}^*_1$ and $\bar{u}^*_2$ and, in consequence, by means of equations S8, S9 and S10, also $\bar{w}^{\text{rec},*}_1$, $\bar{w}^{\text{rec},*}_2$, $\bar{w}^{\text{ff},*}_{1\text{A}}$, $\bar{w}^{\text{ff},*}_{1\text{B}}$, $\bar{w}^{\text{ff},*}_{2\text{A}}$, $\bar{w}^{\text{ff},*}_{2\text{B}}$ and $\bar{u}^*_{\text{inh}}$.

SECTION C: STABILITY

The stability of an equilibrium is determined by the sign of the eigenvalue with the largest real part of the system's Jacobi matrix evaluated at the equilibrium. The nonzero terms of the Jacobi matrix are ($i \in \{1, 2\}$, $k \in \{\text{S1}, \text{S2}\}$):

$$\frac{\partial\dot{\bar{u}}_i}{\partial\bar{u}_i} = -\frac{1}{\tau} + R\bar{n}^{\text{rec}}_i\bar{w}^{\text{lat}}_i\frac{\partial\bar{F}_i}{\partial\bar{u}_i}, \qquad \frac{\partial\dot{\bar{u}}_i}{\partial\bar{u}_{\text{inh}}} = Rw_{i,\text{inh}}\frac{\partial\bar{F}_{\text{inh}}}{\partial\bar{u}_{\text{inh}}},$$

$$\frac{\partial\dot{\bar{u}}_i}{\partial\bar{w}^{\text{rec}}_i} = R\bar{n}^{\text{rec}}_i\bar{F}_i, \qquad \frac{\partial\dot{\bar{u}}_i}{\partial\bar{w}^{\text{ff}}_{ik}} = R\bar{n}^{\text{ff}}_i\bar{I}_k,$$

$$\frac{\partial\dot{\bar{u}}_{\text{inh}}}{\partial\bar{u}_i} = R_{\text{inh}}w_{\text{inh},i}N_i\frac{\partial\bar{F}_i}{\partial\bar{u}_i}, \qquad \frac{\partial\dot{\bar{u}}_{\text{inh}}}{\partial\bar{u}_{\text{inh}}} = -\frac{1}{\tau_{\text{inh}}},$$

$$\frac{\partial\dot{\bar{w}}^{\text{rec}}_i}{\partial\bar{u}_i} = \mu^{\text{rec}}\frac{\partial\bar{F}_i}{\partial\bar{u}_i}\left(2\bar{F}_i - \frac{(\bar{w}^{\text{rec}}_i)^2}{\kappa^{\text{rec}}}\right), \qquad \frac{\partial\dot{\bar{w}}^{\text{rec}}_i}{\partial\bar{w}^{\text{rec}}_i} = \frac{2\mu^{\text{rec}}}{\kappa^{\text{rec}}}(F^{\text{T}} - \bar{F}_i)\bar{w}^{\text{rec}}_i,$$

$$\frac{\partial\dot{\bar{w}}^{\text{ff}}_{ik}}{\partial\bar{u}_i} = \mu^{\text{ff}}\frac{\partial\bar{F}_i}{\partial\bar{u}_i}\left(\bar{I}_k - \frac{(\bar{w}^{\text{ff}}_{ik})^2}{\kappa^{\text{ff}}}\right), \qquad \frac{\partial\dot{\bar{w}}^{\text{ff}}_{ik}}{\partial\bar{w}^{\text{ff}}_{ik}} = \frac{2\mu^{\text{ff}}}{\kappa^{\text{ff}}}(F^{\text{T}} - \bar{F}_i)\bar{w}^{\text{ff}}_{ik}$$

with

$$\frac{\partial\bar{F}_i}{\partial\bar{u}_i} = \beta\bar{F}_i\left(1 - \frac{\bar{F}_i}{\alpha}\right) \quad \text{and} \quad \frac{\partial\bar{F}_{\text{inh}}}{\partial\bar{u}_{\text{inh}}} = \beta\bar{F}_{\text{inh}}\left(1 - \frac{\bar{F}_{\text{inh}}}{\alpha}\right).$$

The eigenvalues of the resulting matrix are determined numerically.

SECTION D: FEED-FORWARD SYNAPTIC WEIGHT CHANGE

For constant pre- and post-synaptic activities (Figure 5 D), Equation 10 can be solved analytically by separation of variables. The resulting time-course $w^{\text{ff}}_i(t)$ depends on the given parameters and initial

conditions:

$$\bar{w}_i^{\mathrm{ff}}(t) = \begin{cases} \bar{w}_i^{\mathrm{ff},*} \coth\left(\sqrt{\frac{\bar{F}_i \bar{I}_i (\bar{F}_i - F_{\mathrm{T}})}{\kappa^{\mathrm{ff}}}}(t-t_0)\mu^{\mathrm{ff}} + \operatorname{arcoth}\left(\frac{\bar{w}_i^{\mathrm{ff}}(t_0)}{w_i^{\mathrm{ff},*}}\right)\right) & \text{for } \bar{w}_i^{\mathrm{ff}}(t_0) > w_i^{\mathrm{ff},*} \wedge \bar{F}_i^{\mathrm{ff}} > F_{\mathrm{T}} \wedge \bar{I}_i > 0, \\ \bar{w}_i^{\mathrm{ff},*} \tanh\left(\sqrt{\frac{\bar{F}_i \bar{I}_i (\bar{F}_i - F_{\mathrm{T}})}{\kappa^{\mathrm{ff}}}}(t-t_0)\mu^{\mathrm{ff}} + \operatorname{artanh}\left(\frac{\bar{w}_i^{\mathrm{ff}}(t_0)}{w_i^{\mathrm{ff},*}}\right)\right) & \text{for } \bar{w}_i^{\mathrm{ff}}(t_0) < w_i^{\mathrm{ff},*} \wedge \bar{F}_i^{\mathrm{ff}} > F_{\mathrm{T}} \wedge \bar{I}_i > 0, \\ \bar{w}_i^{\mathrm{ff},*} \tan\left(\sqrt{\frac{\bar{F}_i \bar{I}_i (\bar{F}_i - F_{\mathrm{T}})}{\kappa^{\mathrm{ff}}}}(t-t_0)\mu^{\mathrm{ff}} + \arctan\left(\frac{\bar{w}_i^{\mathrm{ff}}(t_0)}{w_i^{\mathrm{ff},*}}\right)\right) & \text{for } \bar{w}_i^{\mathrm{ff}}(t_0) < w_i^{\mathrm{ff},*} \wedge \bar{F}_i^{\mathrm{ff}} < F_{\mathrm{T}} \wedge \bar{I}_i > 0, \\ \left(\frac{1}{\bar{w}_i^{\mathrm{ff}}(t_0)} - \frac{F^{\mathrm{T}} - \bar{F}_i^{\mathrm{ff}}}{\kappa^{\mathrm{ff}}}(t-t_o)\mu^{\mathrm{ff}}\right)^{-1} & \text{for } \bar{F}_i^{\mathrm{ff}} = 0 \vee \bar{I}_i = 0 \end{cases}$$

with

$$\bar{w}_i^{\mathrm{ff},*}(\bar{F}_i^*) = \sqrt{\frac{\kappa^{\mathrm{ff}} \bar{F}_i^* \bar{I}}{F^{\mathrm{T}} - \bar{F}_i^*}}.$$

SECTION E: COMPARISON OF BIFURCATION CURVE WITH NETWORK SIMULATION

When comparing the equilibrium structure of the population model dependent on the input amplitude (bifurcation parameter) with the equilibria reached in network simulations (Figure 5 C), the network simulations are initialized close to the different expected stable configurations. For every input amplitude I, we perform two simulations with different initial conditions:

- $w_{ij}^{\mathrm{rec}} = 0.25\hat{w}^{\mathrm{rec}}$ for all realized recurrent synapses and $w_{ij}^{\mathrm{ff}} = \hat{w}^{\mathrm{ff}}$ for all realized feed-forward synapses.
- $w_{ij}^{\mathrm{rec}} = \hat{w}^{\mathrm{rec}}$ for synapses in between 121 neurons in a circle-shaped population, $w_{ij}^{\mathrm{rec}} = 0.25\hat{w}^{\mathrm{rec}}$ for all other realized recurrent synapses and $w_{ij}^{\mathrm{ff}} = \hat{w}^{\mathrm{ff}}$ for all realized feed-forward synapses.

In each case, the network is simulated for $50,000$ s. Every simulation is repeated 50 times with a different random connectivity. To avoid simulation artifacts related to absolute silence of input channels, we assume a small background activity of 0.1α for inactive inputs. In the final state, we either consider all neurons with activity higher than 0.5α or, if there are none, 120 neurons centred around the activity center of the network as population 1. Population 2 is defined as the circular group of 120 neurons with the highest distance (respecting the periodic boundary conditions) to population 1. Within these two populations, we evaluate the mean recurrent weights.

Input Amplitudes: In the network simulation, the functional role of the inhibitory population is two-fold: On the one hand, inhibition mediates the competition between different populations. This role is also captured by the population model. On the other hand, it prevents an active cell assembly from growing without limit by inhibiting neighbouring neurons. This aspect is not reflected in the population model as in the latter the size of the populations is approximated as being fixed. Due to this discrepancy, the population model predicts equilibria also for very large input amplitudes while in the network simulation these input amplitudes lead to full activation of the complete network.

SECTION F: ON THE CAPACITY LIMIT OF THE NETWORK

We discussed in the main text, that the HAs are not overlapping in our current circuit model. Considering an average HA size of 120 neurons and an ideal use of the 900 neurons in the network, we can calculate the theoretical capacity limit of $\lfloor 900/120 \rfloor = 7$ HAs. However, the circular shape of the HA will not allow for an ideal packing density and we draw from our main investigations, that the allocation of HAs is mainly

determined by the symmetry breaking conditions in the initial condition of a learning process (see main text). The determination of the circuit's capacity limit would require a large number of simulations that include multiple memorization processes. In order to learn how HA allocation will function for more than two stimuli and when the circuit will break down, we considered nine input patterns each consisting of four input neurons. We presented the 9 stimuli each like described in the main article, i.e. 10 repetitions for 5s with 1s pause. The resulting weight distributions before (test 0) and after each learning phase (tests 1-9) are depicted in Figures S6 and S7. *Test 0* shows that the initialization of the circuit bears no obvious patterns in the feed-forward and homogeneous recurrent weights. The first 4 learning phases yield expected results of 4 different circular HAs that do not overlap. HA interaction occurs for the first time while learning the 5th stimulus (see *test 5*). HA5 recruits most of the neurons that were originally in HA1, which is left with a small number of high recurrent connections (*HA1*). However, we cannot say that HA5 and HA1 overlap, since it is not a double assignment of neurons to both HAs. The stimulus specific feed-forward weights on HA5 are high for stimulus 5 only and the formerly high weights from stimulus 1 were depressed (data not shown). The small leftover of HA1 is still strongly connected to stimulus 1, though (not shown). *Test 6* shows a similar interaction of HA6 and HA3 although HA6 recruits only a small fraction of HA3. Again, stimulus specific feed-forward weights reveal that it is not an overlap but rather a capture or re-assignment of neurons to a new and exclusive memory representation (not shown). At this point, the network is filled with nearly 5 fully formed HAs and leaves not much 'space' for an average sized circular HA without interacting with existing HAs. Indeed, also in the next learning phase, stimulus 7 is allocating its representation onto HA4 (see *test 7*) and captures nearly all of its neurons. In turn, HA7 is largely captured by HA8 in the following learning phase and HA9 will capture HA8 completely further on. Interestingly, we see that over the course of learning additional stimuli, the old HAs loose their strong recurrent weights. Most prominently we can observe it during the last three learning phases (see *test 7, 8, 9*). This forgetting of old memory representation may be prevented by diverse consolidation mechanisms (Clopath et al., 2008; Tetzlaff et al., 2013).

SECTION G: ON STIMULUS VARIATIONS

The main text discussed the reliability of memory recall with respect to stimulus variations only in the framework of two memorized stimuli. This implies that two HAs compete with each other about which will be activated by the recall stimulus (Figure 2C). Here, we consider the recall dynamics given that the circuit has stored only one HA, to address two different recall protocols: (1) variation of stimulus disparity (like in Figure 2C) to investigate noise robustness and (2) variation of stimulus size. The results are shown in Figure S8 (A, B, respectively), where the response to a stimulus is measured in *active* neurons, that is neurons above $0.5 \cdot \alpha_{rec}$. Each stimulus variation is compared to the circuit's response related to the specific original learning stimulus and the resulting overlap i of active neurons being active in both cases is shown in green. We show data of $n = 10$ individual trials.

In the first case we present noisy versions of the original stimulus; thus, a higher level of disparity indicates a noisier stimulus. In general, we observe that major parts of the HA are responsive to a wide range of disparity levels and a sharp drop to close-to-none response at about 50% disparity. This is in accordance with our expectations towards the pattern completion characteristics of an HA after storing two HAs (see main text). The sudden non-responsiveness means that the existing HA is not favorable over the remaining neurons in network. Thus, probably the alienated stimulus will initiate the formation of a second HA similar to what we described in the main text.

In the second protocol - varied stimulus sizes - we present a partial stimulus of the original one to the system and tested the response for different sizes. An intuitive interpretation of the protocol is partially

occluded or extended variants of the original stimulus (see schematics above the data depiction). We observe close to no response for small stimuli up to half of the original stimulus' size followed by a shard onset of most of the original HA, which converges towards the response levels of the original stimulus. So far the data resembles the case of stimulus disparity and indicates a general pattern completion limit at around 50% of stimulus disparity or similarity. Further extension of stimulus size keeps this level and does not increase the size of response.

SECTION H: COMPARISON WITH BCM PLACTICITY RULE

In the main text our model is based on the combination of Hebbian synaptic plasticity and synaptic scaling. In the following, we focus on the BCM rule (Bienenstock et al., 1982), which is widely-used in diverse learning contexts (e.g. reviewed in Cooper and Bear, 2012). We used the BCM rule as formalized in Dayan and Abbott (2001) such that the corresponding set of differential equations for the dynamics of the synaptic weights and the sliding thresholds $\Theta^{\mathrm{ff/rec}}$ are the following:

$$\frac{\mathrm{d}w_{\mathrm{i,k}}^{\mathrm{ff}}}{\mathrm{d}t} = \mu^{\mathrm{ff}} \cdot F_{\mathrm{i}} I_{\mathrm{k}} \left(F_{\mathrm{i}} - \Theta_{\mathrm{i}}^{\mathrm{ff}} \right) ,$$
$$\frac{\mathrm{d}\Theta_{\mathrm{i}}^{\mathrm{ff}}}{\mathrm{d}t} = \mu_{\Theta}^{\mathrm{ff}} \cdot \left(F_{\mathrm{i}}^{2} - \Theta_{\mathrm{i}}^{\mathrm{ff}} \right) ,$$

$$\frac{\mathrm{d}w_{\mathrm{i,k}}^{\mathrm{rec}}}{\mathrm{d}t} = \mu^{\mathrm{rec}} \cdot F_{\mathrm{i}} F_{\mathrm{k}} \left(F_{\mathrm{i}} - \Theta_{\mathrm{i}}^{\mathrm{rec}} \right) ,$$
$$\frac{\mathrm{d}\Theta_{\mathrm{i}}^{\mathrm{rec}}}{\mathrm{d}t} = \mu_{\Theta}^{\mathrm{ff}} \cdot \left(F_{\mathrm{i}}^{2} - \Theta_{\mathrm{i}}^{\mathrm{rec}} \right) .$$

We presented the stimulus protocol, we applied in the main text, using the same parameter values. For the two new parameters $\mu_{\Theta}^{\mathrm{ff}}$ and $\mu_{\Theta}^{\mathrm{rec}}$, the time constant of the sliding threshold's metaplasticity, we chose a common relative factor c such that $\mu_{\Theta}^{\mathrm{ff}} = c \cdot \mu^{\mathrm{ff}}$ and $\mu_{\Theta}^{\mathrm{rec}} = c \cdot \mu^{\mathrm{rec}}$. Since the dynamics of the sliding threshold has to be faster than the synaptic dynamics to guarantee non-divergent network dynamics (Yger and Gilson, 2015), we choose c equals 10 or 20. Figures S9 & S10 show excerpts from the first learning phase at t = 5s, 15s, 25s and 50s. We see that for $c = 10$ as well as $c = 20$ neither significant activation, nor significant synaptic weight changes (feed forward or recurrent) can be observed. We spared the second learning phase as the dynamics are similar.

REFERENCES

Bienenstock, E. L., Cooper, L. N., and Munro, P. W. (1982). Theory for the development of neuron selectivity: orientation specificity and binocular interaction in visual cortex. *J. Neurosci.* 2, 32–48

Clopath, C., Ziegler, L., Vasilaki, E., Büsing, L., and Gerstner, W. (2008). Tag-trigger-consolidation: a model of early and late long-term potentiation and depression. *PLoS Comput. Biol.* 4, e10000248

Cooper, L. N. and Bear, M. F. (2012). The bcm theory of synapse modification at 30: interaction of theory with experiment. *Nat. Rev. Neurosci.* 13, 798–810

Dayan, P. and Abbott, L. F. (2001). *Theoretical Neuroscience: Computational and mathematical modeling of neural systems* (London: MIT Press)

Rubinov, M. and Sporns, O. (2010). Complex network measures of brain connectivity: uses and interpretations. *Neuroimage* 52, 1059–1069. doi:https://doi.org/10.1016/j.neuroimage.2009.10.003

Tetzlaff, C., Kolodziejski, C., Timme, M., Tsodyks, M., and Wörgötter, F. (2013). Synaptic scaling enables dynamically distinct short- and long-term memory formation. *PLoS Comput Biol* 9(10), e1003307. doi:https://doi.org/10.1371/journal.pcbi.1003307

Yger, P. and Gilson, M. (2015). Models of metaplasticity: a review of concepts. *Front. Comp. Neurosci.* 9, 138

SECTION G: SUPPLEMENTARY FIGURES

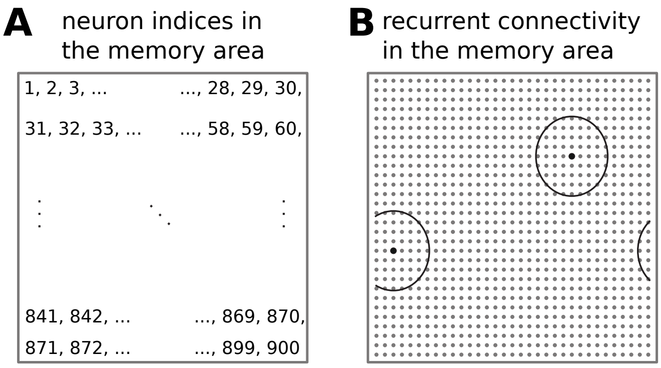

Figure S1. Indexing of neurons in sub-plots and topology. Indexing of neurons and recurrent connectivity in the memory area. (A): The neurons are arranged on a 30x30-grid with indices running from left-top to right-bottom such that each dot in sub-plots of Figure 3 and Figure 4 indicate properties of one neuron. (B): Each neuron is connected to its neighbours, if the position of the neighbouring neuron is within a circle of radius 4 (measured in neuronal units; see two circles as examples). Periodic boundary conditions are introduced to avoid boundary and finite-size effects.

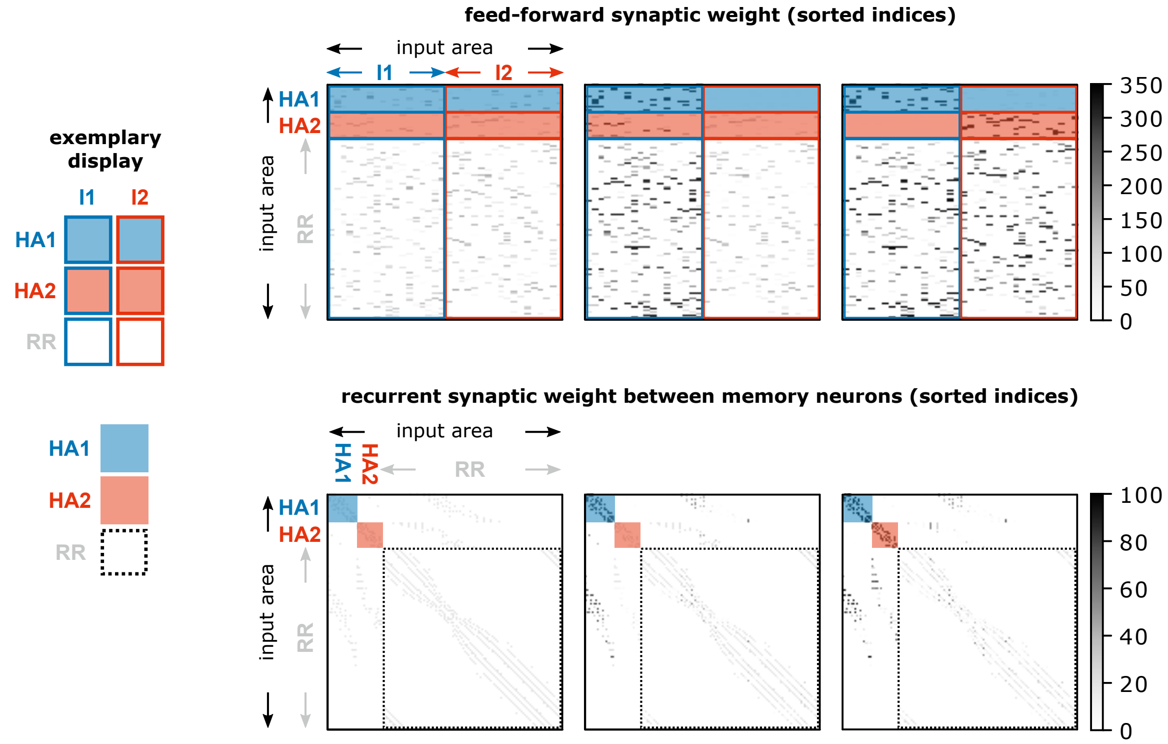

Figure S2. All synaptic weights of feed-forward and recurrent connections. Raw data of the weights of (top) feed-forward and (bottom) recurrent synapses (left) before learning, (middle) after the first learning phase, and (right) after the second learning phase for one system initiation. Indices of neurons in the memory area are sorted according to formed HAs (blue and red shading) while indices of neurons in the input area are sorted according to their input-affiliation (blue and red boxes).

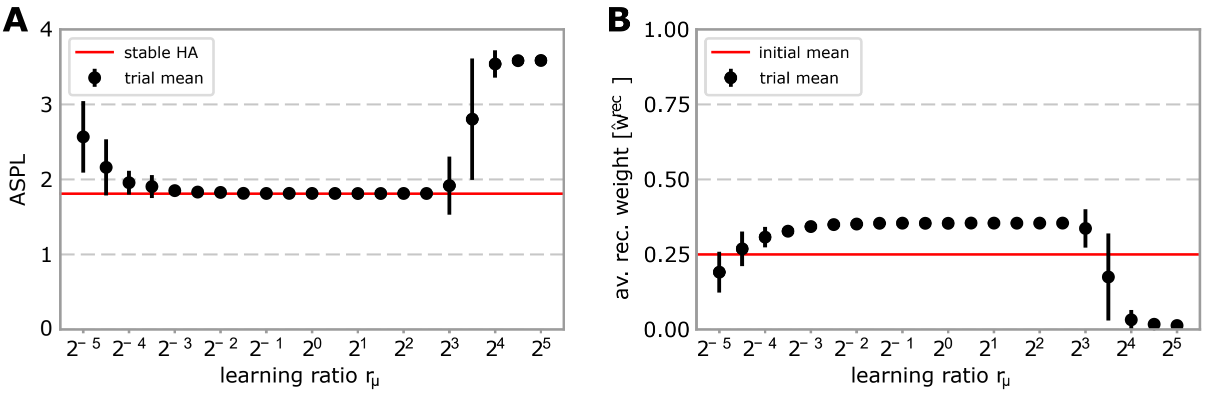

Figure S3. The robustness of HA formation according to parameter variations. The formation of a cell assembly is robust against changes in the velocity of synaptic adaptations of the feed-forward (μ^{ff}; Equation 5) and of the recurrent synapses (μ^{rec}; Equation 6). Given one learning phase, (A) ASPL (between the 10 mostly active neurons) as well as (B) average recurrent synaptic weights within the HA indicate that a HA is formed for a wide range of time scale ratios ($r_\mu = \mu^{\mathrm{rec}}/\mu^{\mathrm{ff}}$). Please note that a low ASPL (≈ 1.8; A, red line) and a significant increase of the average recurrent synaptic weight above the initial mean value (0.25; B, red line) indicate a proper formation of a HA. In the main text, synaptic changes of both types of synapses (feed-forward as well as recurrent) occur with the same time scale $\mu^{\mathrm{ff}} = \mu^{\mathrm{rec}} = \mu$.

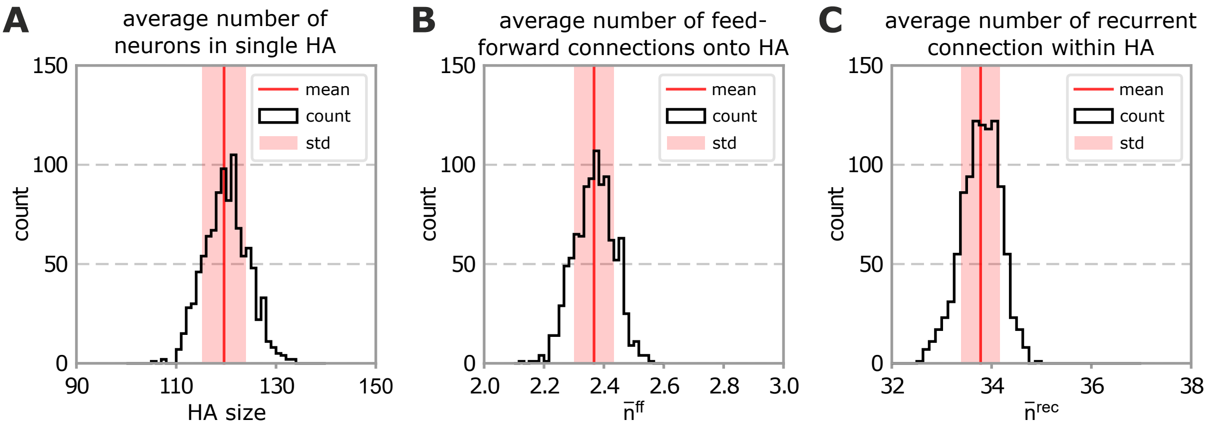

Figure S4. Parameter estimation for population model. Some parameters of the population model are estimated from the full network model. Each panel shows the histogram of a parameter from 1000 different network initializations. Average values are given as *mean±standard deviation*. (A): numbers of neurons within the first formed HA, with average $\bar{N}^{\mathrm{HA}} = 120 \pm 4$. (B): average number of feed-forward connections per HA-neuron from the active input population I1 to corresponding HA, with average $\bar{n}^{\mathrm{ff}} = 2.37 \pm 0.07$. (C): average number of recurrent connections each neuron within the first formed HA receives from other neurons in the same HA, with average $\bar{n}^{\mathrm{rec}} = 33.8 \pm 0.4$).

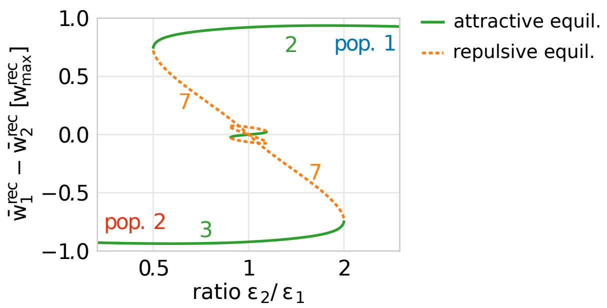

Figure S5. Excitability bifurcation diagram as obtained from the population model. Excitability bifurcation diagram as obtained from the population model. Note that a higher value of ϵ_i means a lower excitability of population i and vice-versa. For a given ratio $\frac{\epsilon_2}{\epsilon_1}$, the used values are $\epsilon_1 = \epsilon_0 \cdot \sqrt{\epsilon_2/\epsilon_1}^{-1}$ and $\epsilon_2 = \epsilon_0 \cdot \sqrt{\epsilon_2/\epsilon_1}$.

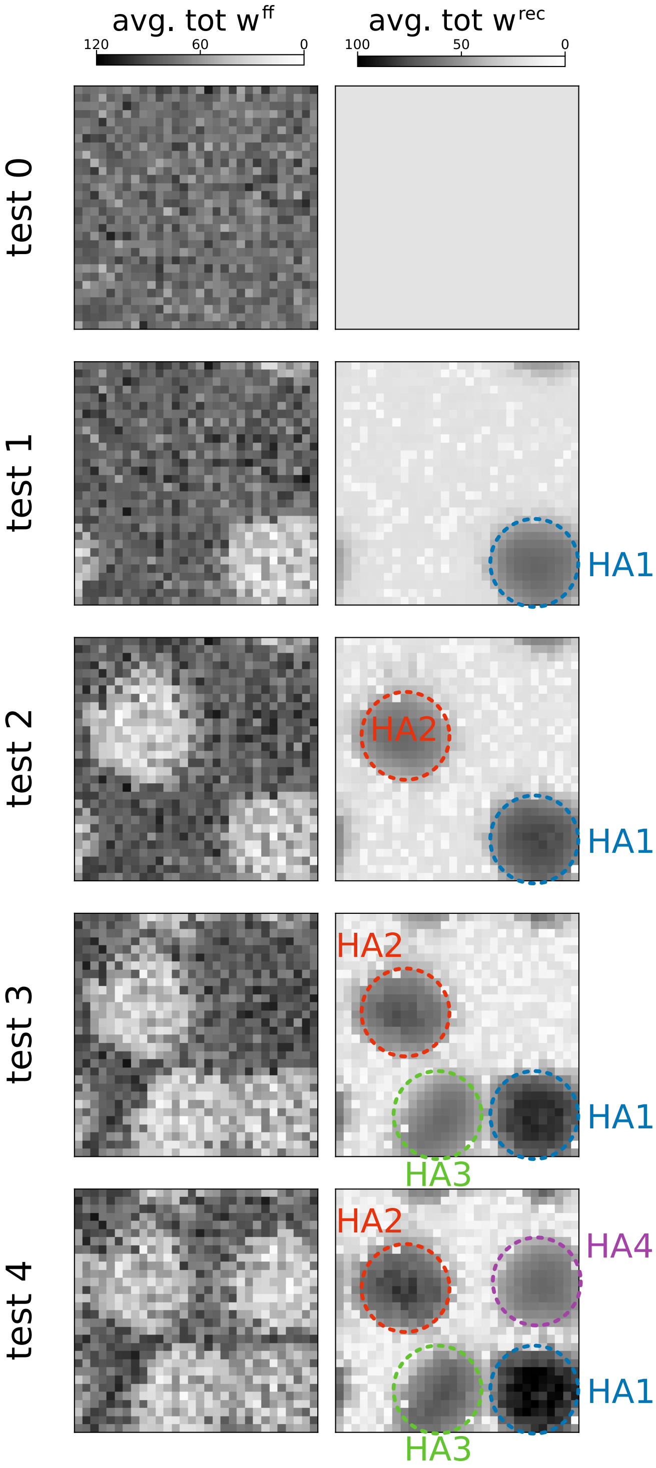

Figure S6. Testing capacity limit - tests 0 to 4. For details see section F.

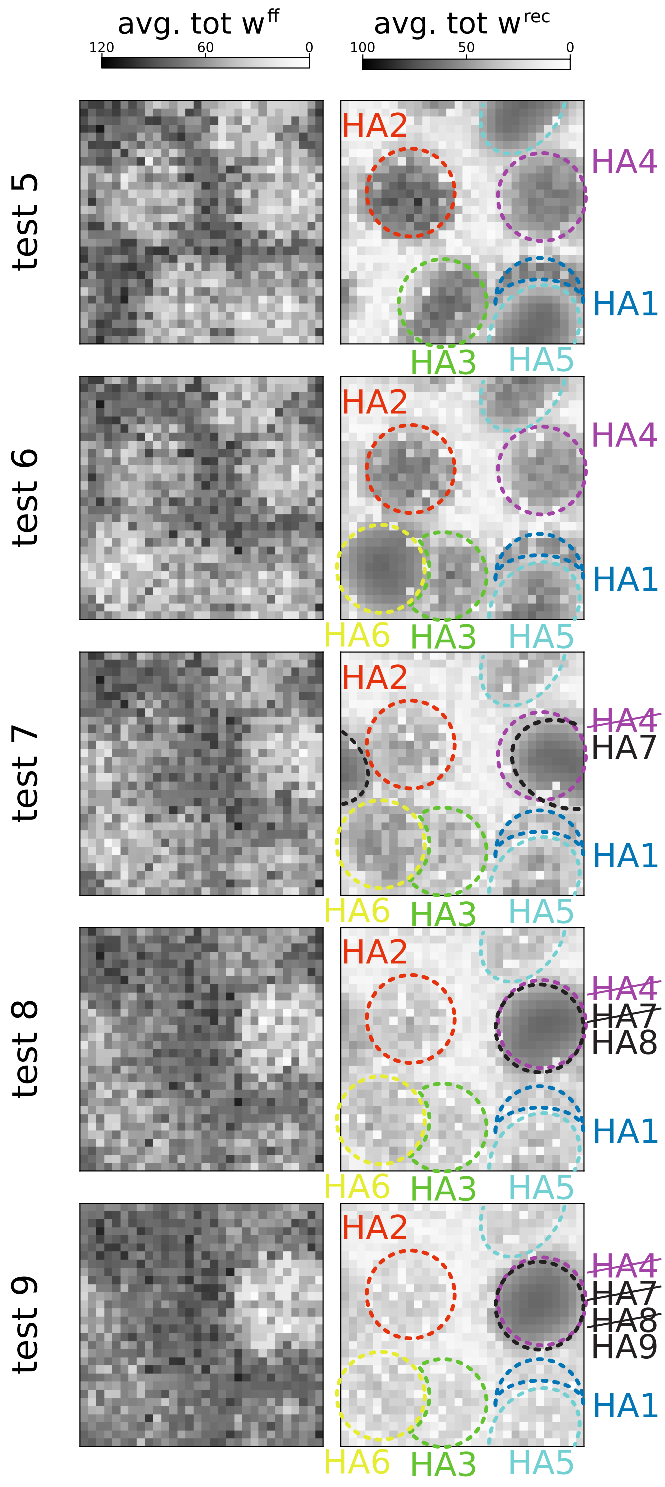

Figure S7. Testing capacity limit - tests 5 to 9. For details see section F.

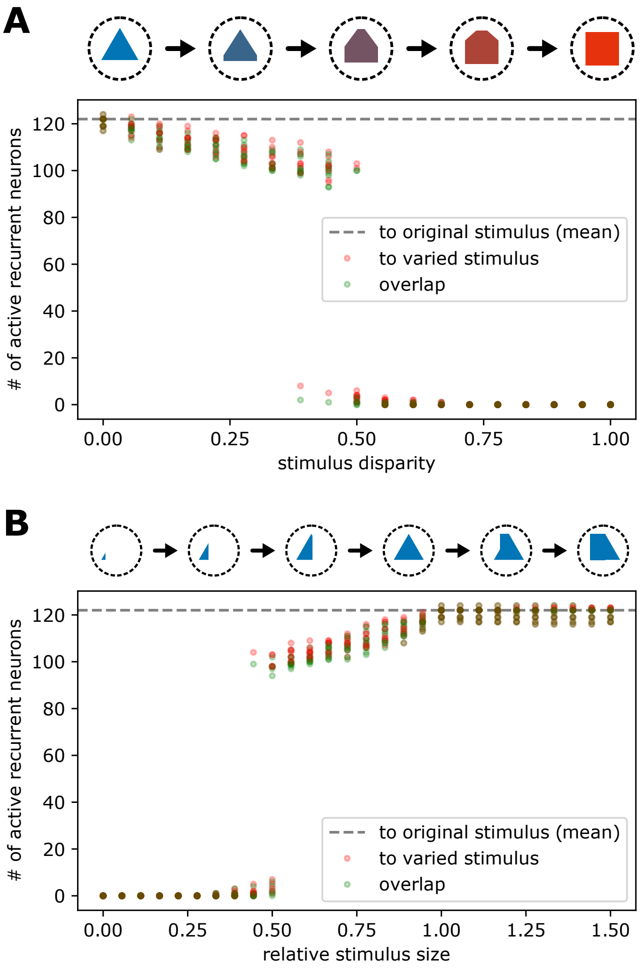

Figure S8. Variation of stimulus disparity & size. For details see section G.

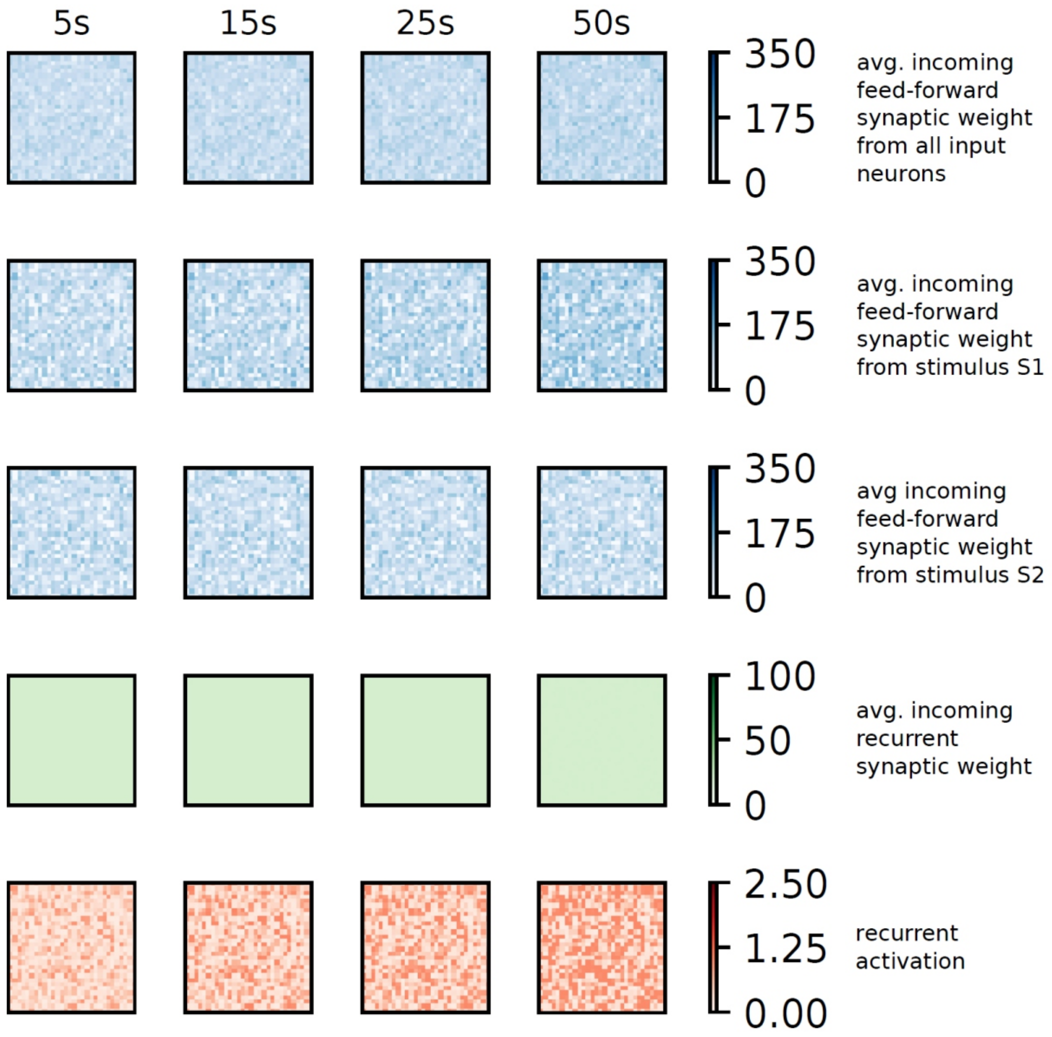

Figure S9. Dynamics given the BCM rule. Color bar scaling equal to the one in Figure 3, except for last row (maximum had to be adapted for better visibility of small values). For details see section H. $c = 10$.

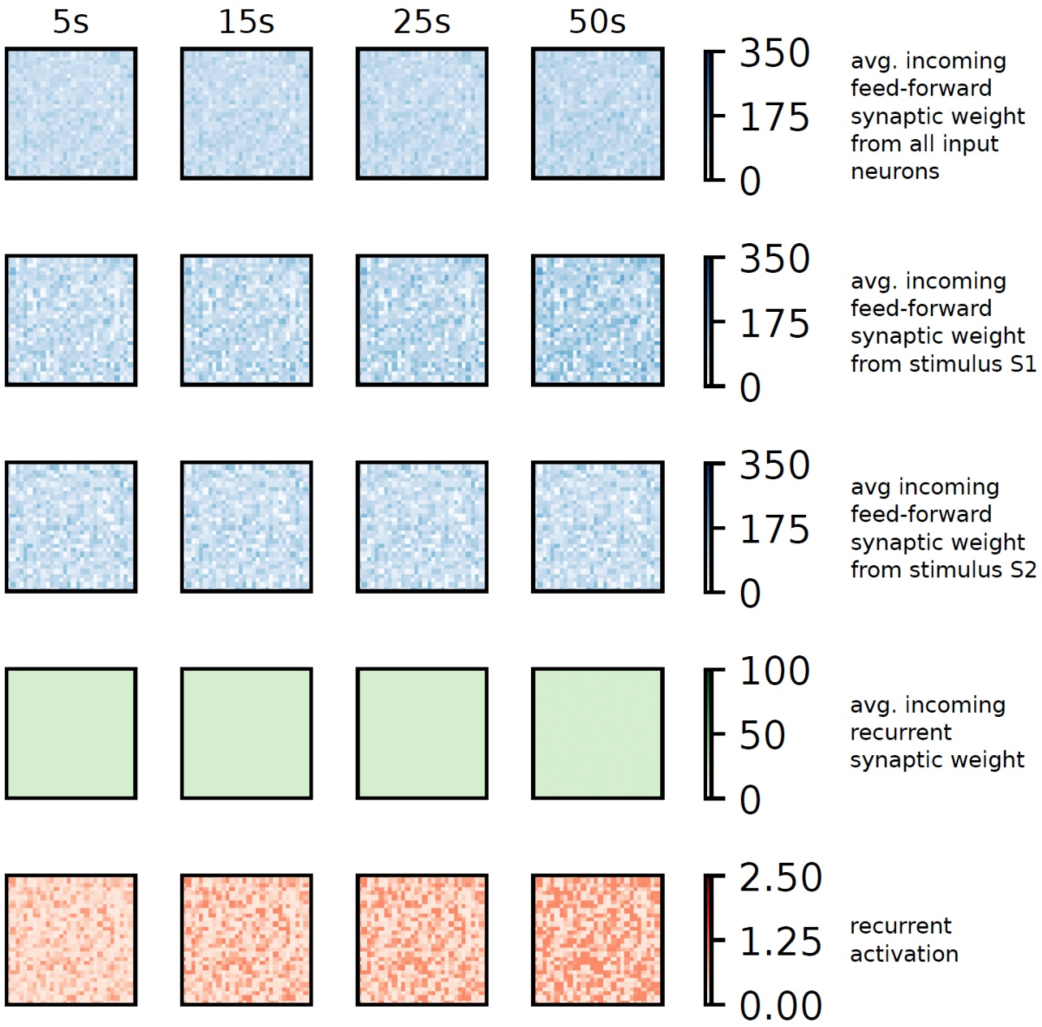

Figure S10. Dynamics given the BCM rule. Color bar scaling equal to the one in Figure 3, except for last row (maximum had to be adapted for better visibility of small values). For details see section H. $c = 20$.
